# Six complete mitochondrial genomes of mayflies from three genera of Ephemerellidae (Insecta: Ephemeroptera) with inversion and translocation of *trnI* rearrangement and their phylogenetic relationships

**DOI:** 10.7717/peerj.9740

**Published:** 2020-08-19

**Authors:** Xiao-Dong Xu, Yi-Yang Jia, Si-Si Cao, Zi-Yi Zhang, Kenneth B. Storey, Dan-Na Yu, Jia-Yong Zhang

**Affiliations:** 1 College of Chemistry and Life Science, Zhejiang Normal University, Jinhua, Zhejiang Province, China; 2 Department of Biology, Carleton University, Ottawa, Canada; 3 Key Lab of Wildlife Biotechnology, Conservation and Utilization of Zhejiang Province, Zhejiang Normal University, Jinhua, Zhejiang, China

**Keywords:** Mitochondrial genome, Ephemerellidae, Phylogenetic relationship, Gene rearrangement, Intergenic regions

## Abstract

As a small order of Pterygota (Insecta), Ephemeroptera has almost 3,500 species around the world. Ephemerellidae is a widely distributed common group of Ephemeroptera. However, the relationship among Ephemerellidae, Vietnamellidae and Teloganellidae is still in dispute. In this study, we sequenced six complete mitogenomes of three genera from Ephemerellidae (Insecta: Ephemeroptera): *Ephemerella* sp. Yunnan-2018, *Serratella zapekinae*, *Serratella* sp. Yunnan-2018, *Serratella* sp. Liaoning-2019, *Torleya grandipennis* and *T. tumiforceps*. These mitogenomes were employed to reveal controversial phylogenetic relationships among the Ephemeroptera, with emphasis on the phylogenetic relationships among Ephemerellidae. The lengths of the six mayfly mitogenomes ranged from 15,134 bp to 15,703 bp. Four mitogenomes of *Ephemerella* sp. Yunnan-2018, *Serratella zapekinae*, *Serratella* sp. Yunnan-2018 and *Serratella* sp. Liaoning-2019 had 22 tRNAs including an inversion and translocation of *trnI*. By contrast, the mitogenomes of *T. tumiforceps* and *T. grandipennis* had 24 tRNAs due to an extra two copies of inversion and translocation of* trnI*. Within the family Ephemerellidae, disparate gene rearrangement occurred in the mitogenomes of different genera: one copy of inversion and translocation *trnI* in the genera *Ephemerella* and *Serratella*, and three repeat copies of inversion and translocation of *trnI* in the genus *Torleya*. A large non-coding region (≥200 bp) between *trnS1* (AGN) and *trnE* was detected in *T. grandipennis* and *T. tumiforceps*. Among the phylogenetic relationship of the Ephemeroptera, the monophyly of almost all families except Siphlonuridae was supported by BI and ML analyses. The phylogenetic results indicated that Ephemerellidae was the sister clade to Vietnamellidae whereas Teloganellidae was not a sister clade of Ephemerellidae and Vietnamellidae.

## Introduction

Ephemeroptera has about 3,500 species around the world, which is a small order of Pterygota (Salles et al., 2018). Having significant roles among freshwater fauna, they are found in many kinds of microhabitats and function in various trophic roles (Salles et al., 2018; Jacobus, Macadam & Sartori, 2019). Considerable effort has been devoted to discovering the phylogenetic relationships among the Ephemeroptera families based on morphology (McCafferty & Edmunds, 1979; McCafferty, 1991; Kluge, 2004), molecular evidence (Ogden & Whiting, 2005), and combined data (Ogden et al., 2009). Despite this, the higher-level phylogeny of mayflies is still a controversial issue (McCafferty & Edmunds Jr, 1979; McCafferty, 1991; Kluge, 2004; Ogden & Whiting, 2005; Ogden et al., 2009), particularly the phylogenetic relationships within the Ephemerellidae, Teloganellidae and Vietnamellidae. Data on morphological characteristics supported a close phylogenetic relationship between Teloganellidae and Vietnamellidae, with the two families forming a sister group to Ephemerellidae (McCafferty, 1991; Kluge, 2004). By contrast, Ephemerellidae was supported as a monophyletic group via phylogenetic trees based on sequences of *12S*, *16S*, *18S*, *28S* and *H3* genes, but the relationships with Teloganellidae and Vietnamellidae remained problematic (Ogden et al., 2009). In addition, Ephemerellidae was supported as the sister clade to Vietnamellidae via phylogenetic analyses based on mitogenomes of Ephemeroptera, whereas Teloganellidae was not a sister clade of Ephemerellidae and Vietnamellidae (Cai et al., 2018; Gao et al., 2018b; Ye et al., 2018; Cao et al., 2020; Xu et al., 2020). Hence, the systematics of Ephemerellidae needed to be clarified by further studies.

The typical mitochondrial genome (mitogenome) of an insect is a circular molecule of 14–20 kb in length including 37 genes (two ribosomal RNA genes, 13 protein-coding genes and 22 transfer RNA genes) along with the control region (CR) (Boore, 1999; Cameron, 2014a). Because of their features including fast evolution rates, small genome sizes, low sequence recombination and maternal inheritance, mitogenomes have been used extensively as molecular markers for phylogenetic analyses and comparative or evolutionary genomic research (Boore, 2006; Cameron, 2014a; Ma et al., 2015; Cheng et al., 2016). Currently, 29 complete (or nearly complete) mitogenomes of Ephemeroptera from thirteen families have been released on NCBI (Zhang et al., 2008; Li, Qin & Zhou, 2014; Tang et al., 2014; Zhou et al., 2016; Cai et al., 2018; Gao et al., 2018b; Ye et al., 2018; Song et al., 2019; Cao et al., 2020; Xu et al., 2020), but there was only one partial mitogenome of Ephemerellidae. These small numbers of mitogenomes have restricted our understanding of the phylogenetic relationships and biogeography of the Ephemerellidae.

Gene rearrangements can be frequently observed in insect mitogenomes (Yukuhiro et al., 2002; Oliveira et al., 2008; Zhang et al., 2008; Dowton et al., 2009; Wan et al., 2012; Leavitt et al., 2013; Wei et al., 2014; Dickey et al., 2015; Cheng et al., 2016; Gao et al., 2018a; Gao et al., 2018b; Zhang et al., 2018a; Zhang et al., 2018b), whereas gene losses (gene deletions) or extra copies (gene duplications) are rarer (Cameron, 2014a). When gene duplication occurs, most extra tRNA copies have been observed near the CR, which supported the idea that gene duplication events are mostly due to replication slippage mechanisms (Macey et al., 1997; Zhang & Hewitt, 1997). The gene organization of most mayfly mitogenomes and the typical insect mitogenome are generally in great accordance, except for some species of Baetidae, Ephemerellidae, Heptageniidae and Siphluriscidae (Zhang et al., 2008; Li, Qin & Zhou, 2014; Tang et al., 2014; Gao et al., 2018b; Song et al., 2019). For instance, *Siphluriscus chinensis* (Siphluriscidae) had one extra *trnK*-like gene (*trnK* 2 (AAA) (Li, Qin & Zhou, 2014). Within the family Heptageniidae, the mitogenomes of *Parafronurus youi*, *Parafronurus* sp. XL-2019, *Epeorus herklotsi*, *Epeorus* sp. JZ-2014, *Epeorus* sp. MT-2014, *Epeorus* sp. XL-2019 and *Rhithrogena* sp. XL-2019 had an extra *trnM (Zhang et al., 2008; Tang et al., 2014; Gao et al., 2018b; Song et al., 2019)*. *Ephemerella* sp. MT-2014 (Ephemerellidae) had an inversion of *trnI* (Tang et al., 2014). Additionally, *Alainites yixiani* (GU479735) (Baetidae) showed a gene arrangement of *trnI*-*trnW*-*trnQ*-*trnY*-*trnM*. Therefore, these different sets of data suggested that specific gene rearrangements may occur in the mitogenomes of different families. The present study is important and original in that it explores the relationships between mitogenome rearrangements and taxonomic categories of Ephemeroptera.

In order to discuss the characteristics of *trnI* inversion in the Family Ephemerellidae and explore the phylogenetic relationships of Ephemerellidae, we sequenced six complete mitogenomes belonging to three genera of Ephemerellidae. The compositional and structural features of these six mitogenomes are described and gene rearrangements were analyzed to explain the mechanism of mitochondrial gene rearrangements.

## Materials and Methods

### Sampling collection and DNA extraction

The voucher specimens of *Ephemerella* sp. Yunnan-2018, *S. zapekinae*, *Serratella* sp. Yunnan-2018, *Serratella* sp. Liaoning-2019, *T. grandipennis* and *T. tumiforceps* were separately captured from Ning’er Yunnan province, Xiuyan Liaoning province, Ning’er Yunnan province, Kuandian Liaoning province, Longquan Zhejiang province and Tonglu Zhejiang province, China, respectively. After morphological identification, the specimens were deposited at −40 °C in the Animal Specimen Museum, College of Life Sciences and Chemistry, Zhejiang Normal University, China. Total genomic DNA was extracted from tissues of each complete specimen by Ezup Column Animal Genomic DNA Purification Kit (Sangon Biotech Company, Shanghai, China). The Animal Research Ethics Committees of Zhejiang Normal University approved the experimental design.

### PCR amplification and sequencing

Several partial fragments were amplified using common primers (Table S1), as described in Simon et al. (1994), Simon et al. (2006), Zhang et al. (2008) and Zhang et al. (2018b). Subsequently, we designed species-specific primers (Table S1) according to sequences previously obtained from universal primers using Primer Premier 5.0 (Lalitha, 2000). Both PCR (product length < 3,000 bp) and Long-PCR (product length >3,000 bp) methods were used separately with *Takara Taq* and *Takara LA Taq* DNA polymerase in a reaction volume of 50 μL. The amplifications for both normal PCR and Long-PCR were performed under the conditions as described in Zhang et al. (2018b). All the products were then sequenced bidirectionally using the primer-walking method and AB13730XL by Sangon Biotech Company (Shanghai, China).

### Mitogenome annotation and sequence analyses

The nucleotide fragments were assembled and analyzed using DNASTAR Package v.7.1 (Burland, 1999). We identified the tRNA genes on the MITOS web server (http://mitos.bioinf.uni-leipzig.de/index.py) (Bernt et al., 2013) using the genetic codes for invertebrate mitogenomes and secondary structures were obtained using a force-directed graph layout (http://rna.tbi.univie.ac.at/forna) (Kerpedjiev, Hammer & Hofacker, 2015). After using Clustal X (Thompson et al., 1997) to compare the genes with homologous sequences of other mayfly mitogenomes, we determined the sequences of the two rRNA genes (*12S* and *16S rRNA*). The *trnI* sequences of *T. grandipennis* and *T. tumiforceps* were aligned using the Clustal W program implemented in Mega 7.0 (Kumar, Stecher & Tamura, 2016). Then, we translated the 13 protein-coding genes using the invertebrate mitogenome genetic code in order to find the open reading frames between tRNAs (Cameron, 2014b). The six mitogenomes were deposited in GenBank with the accession numbers MT274127–MT274132. The mitogenome maps of the six mayfly species were drawn using GenomeVx (http://wolfe.ucd.ie/GenomeVx) (Conant & Wolfe, 2008). Codon usage, A+T content and relative synonymous codon usage (RSCU) of the protein-coding genes were calculated using PhyloSuite (Zhang et al., 2020). Using the following formulas: AT-skew = (A-T)/(A+T); GC-skew = (G-C)/(G+C) (Perna & Kocher, 1995), we calculated the composition skewness. Tandem repeats in these mitogenomes were identified using Tandem Repeat Finder V 4.09 (http://tandem.bu.edu/trf/trf.submit.options.html) (Benson, 1999). Additionally, the secondary structures of tandem repeat units in the non-coding regions were predicted by MFold (Zuker, 2003).

### Phylogenetic methods

Twenty-four formerly published mayfly mitogenomes along with the newly determined sequences (Table 1) were used in the phylogenetic analyses to discuss the relationships of Ephemeroptera (Zhang et al., 2008; Li, Qin & Zhou, 2014; Tang et al., 2014; Zhou et al., 2016; Cai et al., 2018; Gao et al., 2018b; Ye et al., 2018; Cao et al., 2020; Xu et al., 2020). Because long-branch attraction existed in species of Baetidae, we deleted two species of Baetidae and rebuilt the BI and ML phylogenetic trees. The nucleotide sequences of the 13 protein-coding genes were used as the dataset to build the BI and ML phylogenetic trees as in Zhang et al. (2018b). In addition, *Siphluriscus chinensis* acted as the outgroup (Li, Qin & Zhou, 2014). We used Clustal W in the program Mega 7.0 to align each of the 13 protein-coding genes (Kumar, Stecher & Tamura, 2016). Also, we identified the conserved regions using the Gblock 0.91b program (Castresana, 2000). Concatenating the resulting alignments with Geneious 8.1.6 (Kearse et al., 2012), we inferred the optimal partitioning strategy using the program PartitionFinder 1.1.1 (Lanfear et al., 2012) and chose the best model based on the Bayesian Information Criterion (BIC). The best model is listed in Table S2. Each of three codon positions for the 13 protein-coding genes defined the data blocks. On the one hand, we implemented ML analysis in RAxML 8.2.0 with a GTRGAMMAI model. Also, we evaluated branch support for each node with 1,000 replicates (Stamatakis, 2014). On the other hand, we implemented BI analysis in MrBayes 3.2 with the best model estimated (Table S2), which were divided into four chains (three hot and one cold), with a run of 10 million generations in total length and sampling every 1,000 generations (Ronquist et al., 2012). The first 25% of generations were removed as burn-in. After the average standard deviation of split frequencies was below 0.01, we judged that BI analysis had reached sufficient convergence.

**Table 1:** Species of Ephemeroptera used to construct the phylogenetic relationships along with GenBank accession numbers.

Family	Species	Genome length	Number of tRNAs	GenBank No.	References
Ameletidae	*Ameletus* sp. MT-2014	15,141	22	KM244682	Tang et al. (2014)
Baetidae	*Baetis* sp. PC-2010	14,883	22	GU936204	Directly Submitted
Baetidae	*Alainites yixiani*	14,589	22	GU479735	Directly Submitted
Caenidae	*Caenis* sp. YJ-2009	15,351	22	GQ502451	Directly Submitted
Caenidae	*Caenis* sp. JYZ-2018	15,254	22	MG910499	Cai et al. (2018)
Caenidae	*Caenis* sp. JYZ-2020	15,392	22	MN356096	Xu et al. (2020)
Ephemerellidae	*Ephemerella* sp. MT-2014	14,896	22	KM244691	Tang et al. (2014)
Ephemerellidae	*Ephemerella* sp. Yunnan-2018	15,256	22	MT274127	This study
Ephemerellidae	*Serratella zapekinae*	15,703	22	MT274130	This study
Ephemerellidae	*Serratella* sp. Yunnan-2018	15,134	22	MT274129	This study
Ephemerellidae	*Serratella* sp. Liaoning-2019	15,523	22	MT274128	This study
Ephemerellidae	*Torleya grandipennis*	15,330	24	MT274131	This study
Ephemerellidae	*Torleya tumiforceps*	15,599	24	MT274132	This study
Ephemeridae	*Ephemera orientalis*	16,463	23	EU591678	Directly Submitted
Heptageniidae	*Epeorus herklotsi*	15,502	23	MG870104	Gao et al. (2018b)
Heptageniidae	*Epeorus* sp. JZ-2014	15,338	23	KJ493406	Directly Submitted
Heptageniidae	*Epeorus* sp. MT-2014	15,456	23	KM244708	Tang et al. (2014)
Heptageniidae	*Paegniodes cupulatus*	15,715	23	HM004123	Directly Submitted
Heptageniidae	*Parafronurus youi*	15,481	23	EU349015	Zhang et al. (2008)
Isonychiidae	*Isonychia ignota*	15,105	22	HM143892	Directly Submitted
Isonychiidae	*Isonychia kiangsinensis*	15,456	22	MH119135	Ye et al. (2018)
Leptophlebiidae	*Choroterpides apiculata*	15,199	22	MN807287	Cao et al. (2020)
Leptophlebiidae	*Habrophlebiodes zijinensis*	14,355	22	GU936203	Directly Submitted
Potamanthidae	*Potamanthus* sp. MT-2014	14,937	22	KM244674	Tang et al. (2014)
Siphlonuridae	*Siphlonurus immanis*	15,529	22	FJ606783	Directly Submitted
Siphlonuridae	*Siphlonurus* sp. MT-2014	14,745	22	KM244684	Tang et al. (2014)
Siphluriscidae	*Siphluriscus chinensis*	16,616	23	HQ875717	Li, Qin & Zhou (2014)
Teloganodidae	*Teloganodidae* sp. MT-2014	12,435	22	KM244703;	Tang et al. (2014)
		2,817		KM244670	
Vietnamellidae	*Vietnamella dabieshanensis*	15,761	22	HM067837	Directly Submitted
Vietnamellidae	*Vietnamella* sp. MT-2014	15,043	22	KM244655	Tang et al. (2014)

## Results

### Mitogenome organization and composition

We obtained and characterized the complete mitogenomes of *Ephemerella* sp. Yunnan-2018, *S*. *zapekinae*, *Serratella* sp. Yunnan-2018, *Serratella* sp. Liaoning-2019, *T. grandipennis* and *T. tumiforceps.* These were deposited in the GenBank database (Table 1). The six new mitogenomes were double circular DNA molecules with lengths ranging from 15,134 bp to 15,703 bp (Fig. S1). The size variation of the six mitogenomes was large due to different intergenic nucleotides (IGNs), the size of the CR, and the presence of additional copies of *trnI*. The nucleotide compositions of the six mayfly mitogenomes had a high A+T bias between 61.1% and 66.1%. All six mitogenomes showed a negative AT-skew on the majority strand and negative GC-skew as well (Table 2), as occurs in most other Ephemeroptera mitogenomes. The AT-skew values on the majority strand for the six mayfly species ranged from −0.053 (*S. zapekinae*) to −0.017 (*Serratella* sp. Yunnan-2018) whereas the GC-skew for the majority strand ranged from −0.234 (*Ephemerella* sp. Yunnan-2018) to −0.159 (*S. zapekinae*). In addition, we analyzed the sizes and nucleotide compositions of the previously published mayfly mitogenomes. Differences in mitochondrial sequences of Ephemeroptera were mainly determined by different sizes of the CR. Among all sequenced Ephemeroptera mitogenomes (Table 1) (Zhang et al., 2008; Li, Qin & Zhou, 2014; Tang et al., 2014; Zhou et al., 2016; Cai et al., 2018; Gao et al., 2018b; Ye et al., 2018; Cao et al., 2020; Xu et al., 2020), the length of the mitogenome of *S. chinensis* (16,616 bp) was the longest because of the longest CR (1,829 bp) (Li, Qin & Zhou, 2014), whereas that of *Baetis* sp. PC-2010 (GU936204) (14,883 bp) was the shortest CR (340 bp). The AT-skews of mitogenomes on the majority strand were negative ranging from *Baetis* sp. PC-2010 (GU936204) (−0.093) to *Caenis* sp. YJ-2009 (GQ502451) (−0.001) except for positive skews in *Ephemera orientalis* (EU591678) (0.03), *S. chinensis* (0.019) (Li, Qin & Zhou, 2014) and *Ameletus* sp. MT-2014 (0.01) (Tang et al., 2014). The GC-skews of the mitogenomes on the majority strand were also negative for the same strand ranging from *Habrophlebiodes zijinensis* (GU936203) (−0.296) to *S. chinensis* (−0.139) (Li, Qin & Zhou, 2014) except for *Baetis* sp. PC-2010 (GU936204) (0.006), *A. yixiani* (GU479735) (0.14) and *Ameletus* sp. MT-2014 (0.202) (Tang et al., 2014).

**Table 2:** Base composition of six mayfly mitochondrial genomes.

**Species name**	**A+T (%)**				**AT-skew**					**GC-skew**				
	**Mito**	**PCGs**	**rRNAs**	**Non- coding region**	**Mito**	**PCGs-H**	**PCGs-L**	**rRNAs**	**Non- coding region**	**Mito**	**PCGs-H**	**PCGs-L**	**rRNAs**	**Non- coding region**
*Ephemerella* sp. Yunnan-2018	61.1	60.4	65.1	54.5	−0.035	−0.176	−0.231	0.07	−0.536	−0.234	−0.198	0.223	0.304	−0.793
*Serratella zapekinae*	65.2	64.7	68.5	61.9	−0.053	−0.209	−0.217	0.056	−0.248	−0.159	−0.108	0.241	0.22	−0.134
*Serratella* sp. Yunnan-2018	66.1	65.5	68.8	62.1	−0.017	−0.155	−0.217	0.035	−0.186	−0.17	−0.14	0.207	0.245	−0.264
*Serratella* sp. Liaoning-2019	65.2	64.5	67.1	68.5	−0.04	−0.179	−0.226	0.062	−0.234	−0.173	−0.097	0.177	0.229	−0.822
*Torleya grandipennis*	61.2	60.8	63.1	61.1	−0.045	−0.201	−0.222	0.069	−0.212	−0.18	−0.164	0.177	0.291	−0.382
*Torleya tumiforceps*	62.6	62.6	64.3	63.6	−0.051	−0.208	−0.23	0.075	−0.275	−0.165	−0.172	0.196	0.263	−0.231

### Protein-coding genes (PCGs) and codon usages

The sizes of the protein-coding genes in the six mitogenomes were similar to each other. The locations and orientations of the 13 PCGs within the six mitogenomes were identical to those of most mayfly species (Tables S3, S4). The majority of the PCGs within the six mitogenomes began with conventional initiation codons, except TTG that was used for *ND3* (*Ephemerella* sp. Yunnan-2018) and *ND6* (*Ephemerella* sp. Yunnan-2018, *T. tumiforceps*, *T. grandipennis*, *S. zapekinae*, *Serratella* sp. Liaoning-2019) along with GTG assigned to *ND1* (*Serratella* sp. Yunnan-2018), *ND3* (*Serratella* sp. Yunnan-2018), *ND4* (*Serratella* sp. Liaoning-2019), and *ND5* (*S. zapekinae*, *Serratella* sp. Liaoning-2019). Typical stop codons (TAA/TAG) were found in most PCGs, except for incomplete terminal codons, such as T used for *COX2* (*Serratella* sp. Yunnan-2018, *S. zapekinae*), *COX3* (*T. tumiforceps*, *T. grandipennis*), *Cyt b* (*S. zapekinae*, *T. tumiforceps*), *ND4* (*Serratella* sp. Liaoning-2019), *ND5* (*S. zapekinae*, *Serratella* sp. Liaoning-2019, *T. tumiforceps*, *T. grandipennis*) and *ND6* (*Serratella* sp. Liaoning-2019), as well as TA used for *ND4* (*T. tumiforceps*, *T. grandipennis*). Incomplete terminal codons perform a significant function in polycistronic transcription cleavage and polyadenylation processes (Anderson et al., 1981; Ojala, Montoya & Attardi, 1981), which can be commonly observed in Ephemeroptera mitogenomes (Zhang et al., 2008; Li, Qin & Zhou, 2014; Tang et al., 2014; Zhou et al., 2016; Cai et al., 2018; Gao et al., 2018b; Ye et al., 2018; Cao et al., 2020; Xu et al., 2020). The average A+T contents of the 13 PCGs within the six mitogenomes ranged from 60.4% to 65.5% (Table 2). The PCGs of both the majority and minority strands showed negative AT-skews, identical to the 29 other published Ephemeroptera mitogenomes (Zhang et al., 2008; Li, Qin & Zhou, 2014; Tang et al., 2014; Zhou et al., 2016; Cai et al., 2018; Gao et al., 2018b; Ye et al., 2018; Song et al., 2019; Cao et al., 2020; Xu et al., 2020). Interestingly, a negative AT-skew existed in the PCGs on the majority strand of all mayfly mitogenomes, which contradicted the fact that the majority of hexapod species show a positive AT-skew (Perna & Kocher, 1995; Carapelli et al., 2007). This phenomenon indicates that a special phylogeny-related strand asymmetry reverse on the majority strand has appeared in mayfly species (Wei et al., 2010; Li, Qin & Zhou, 2014).

**Figure 1:**
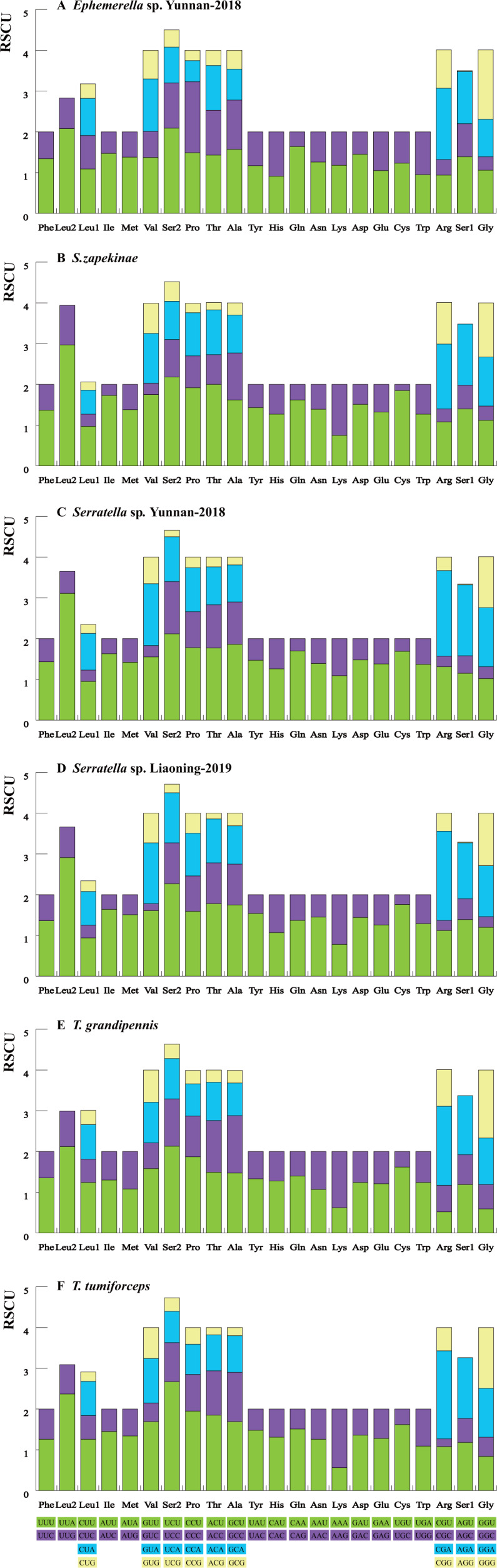
The RSCU of six mayfly mitochondrial genomes. Codon families are provided on the *x*-axis and the different combinations of synonymous codons that code for each amino acid (the RSCU: relative synonymous codon usage) are defined on the *Y*-axis. (A) *Ephemerella* sp. Yunnan-2018, (B) *S. zapekinae*, (C) *Serratella* sp. Yunnan-2018, (D) *Serratella* sp. Liaoning-2019, (E) *T. grandipennis*, (F) *T. tumiforceps*.

We calculated the relative synonymous codon usage (RSCU) of the six mayfly mitogenomes (Fig. 1, Table S5). The analysis showed a higher utilization of A or T nucleotides in the third codon position compared to other synonymous codons, generally regarded as the function of genome bias, optimum choice of tRNA usage or the benefit of DNA repair (Crozier & Crozier, 1993). According to comparative analyses, the most frequently utilized codons were highly similar within the six mayfly species. UUA (Leu), AUU (Ile) and UUU (Phe) were the most habitually used codons (>170) within the PCGs of the six mitogenomes, whereas UGC (Trp), CGC (Arg) and AGG (Ser) were the least used (<20). The strong AT mutation bias manifestly affected the codon usage, which proved that U and A were preferred in codons (Powell & Moriyama, 1997; Rao et al., 2011). Moreover, those AT-rich codons encoded the most plentiful amino acids, e.g., Leu (16.94%-17.47%), which suggested that the AT bias would also affect acid constituents of the proteins encoded by the mitochondrial genes (Foster, Jermiin & Hickey, 1997; Min & Hickey, 2007).

### Transfer RNAs and ribosomal RNAs

**Figure 2:**
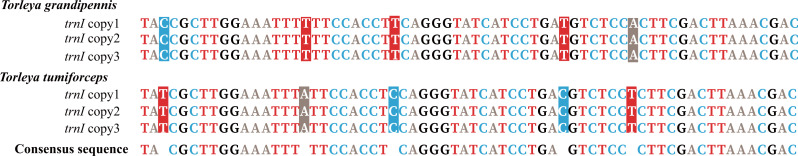
Alignment of *trnI* sequences in *T. tumiforceps* and *T. grandipennis*.

Mitogenomes of *Ephemerella* sp. Yunnan-2018, *S. zapekinae*, *Serratella* sp. Yunnan-2018 and *Serratella* sp. Liaoning-2019 had 22 tRNA genes (Table 1). By contrast, the mitogenomes of *T. grandipennis* and *T. tumiforceps* had 24 tRNA genes (Table 1) including an extra two copies of *trnI*. *Ephemerella* sp. Yunnan-2018, *S. zapekinae*, *Serratella* sp. Yunnan-2018 and *Serratella* sp. Liaoning-2019 shared the translocation of *trnI*, which moved from between the CR and *trnQ* into a position between *12S rRNA* and CR, along with the inversion of *trnI* (Fig. S1A). Furthermore, the mitogenomes of *T. grandipennis* and *T. tumiforceps* had three identical copies of the inversion of *trnI*, all of which were translocated to between *12S rRNA* and CR (Fig. S1B). The lengths of these tRNA genes in the six mayfly mitogenomes ranged from 60 bp to 70 bp. The secondary structures of tRNA genes identified in these mayfly mitogenomes are shown in Figs. S2–S7. All the predicted tRNAs showed the typical clover-leaf secondary structure of mitochondrial tRNAs except for *trnS1* (AGN) in *S*. *zapekinae* (Fig. S3N). Its dihydrouridine (DHU) arm formed a simple loop, which has been observed in many insects (Zhang et al., 2018a; Wang et al., 2019). Quite a few mismatched pairs existed in terms of tRNA secondary structure within all six mayfly mitogenomes, e.g., U-G in the DHU arm and amino acid acceptor arm of *trnQ* and U-G in the T ψC (T) arm of *trnN* and *trnS1* (AGN). In general, these mismatches are fundamental units of tRNA secondary structure and are nearly isomorphic to normal base pairs, as commonly observed in mayflies or other insects (Varani & McClain, 2000; Zhang et al., 2008; Li, Qin & Zhou, 2014; Wang et al., 2019). It has been reported that mismatches may enhance aminoacylation and translation (McClain, 2006). In *T. tumiforceps* and *T. grandipennis*, three copies of *trnI* had the same structure due to the identical nucleotide sequences (Fig. 2). In general, the characteristics of tRNAs were conserved among the six mitogenomes, as is commonly observed in most insects, except for the inversion and translocation of *trnI* rearrangements.

**Figure 3:**
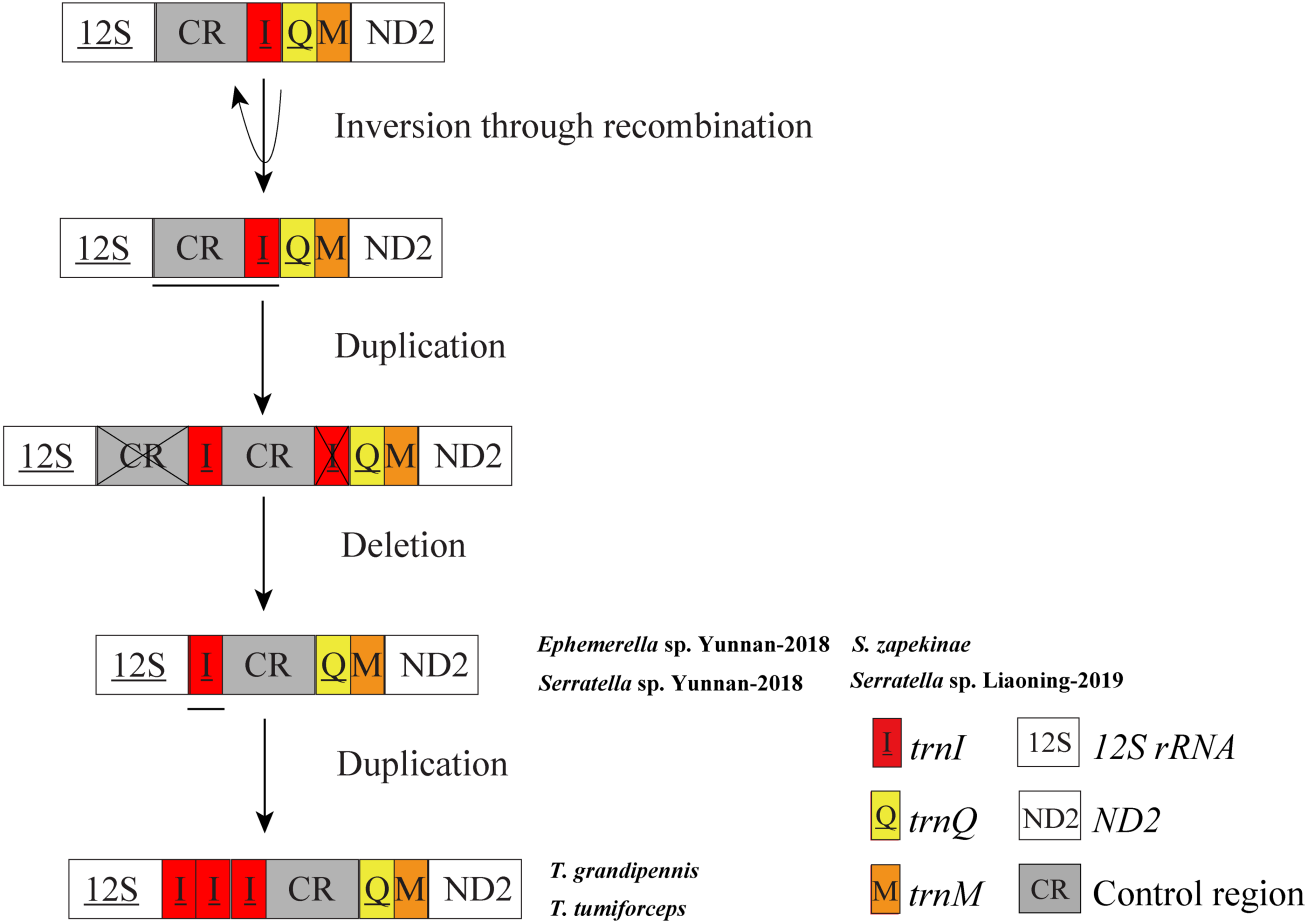
Proposed mechanism of gene arrangements in the six mayfly mitogenomes. Gene sizes are not drawn to scale. For each image, genes encoded by the minority strand are underlined and without underline are encoded by the majority strand. White boxes represent genes with the same relative position as in the ancestral insect arrangement pattern. Horizontal lines and cross symbols represent gene duplications and gene deletions, respectively. Red boxes represent gene inversions. Dark grey boxes represent non-coding regions. The remaining genes and gene orders were identical to the ancestral insect arrangement and are left out. The images show that firstly, trnI was inversed through recombination; secondly, CR-trnI was tandem duplicated; thirdly, the first CR and the second trnI were deleted according to the TDRL model; and fourthly, the inversion and translation of trnI was duplicated three times.

In mitochondrial gene rearrangements of *Ephemerella* sp. Yunnan-2018, *S*. *zapekinae*, *Serratella* sp. Yunnan-2018 and *Serratella* sp. Liaoning-2019, we observed the inversion and translocation of *trnI* which is identical to *Ephemerella* sp. MT-2014 (Tang et al., 2014). We suggest that these rearrangements are presumably caused by the Tandem Duplication and Random Loss (TDRL) model (Moritz, Dowling & Brown, 1987; Arndt & Smith, 1998) and Recombination model (Lunt & Hyman, 1997). A schematic illustration of rearrangement events for these mitogenomes is presented in Fig. 3. The inversion of *trnI* may result from the breaking of the mitogenomes at *trnI* along with recombination of *trnI* on the other strand. The translocation of *trnI* was probably caused by a tandem duplication of the gene block CR-*trnI*, resulting in a CR-*trnI*-CR-*trnI* arrangement, then the first copy of CR and the second copy of *trnI* were subsequently randomly lost. This left the *trnI*-CR arrangement. By contrast to the mentioned rearrangements, *T. tumiforceps* and *T. grandipennis* also had two extra copies of *trnI*, which were the same as the original *trnI*. The presence of three *trnI* copies may have been caused by additional gene duplication *trnI*-*trnI*-*trnI*. Nevertheless, since each rearrangement event was independent, the actual order of events has yet to be determined. It was reported by Boore (1999) that the flanks of the control region were the hot spots for gene rearrangements in Arthropoda, which is in accordance with the site of *trnI* rearrangement in mayflies. As Taylor et al. (1993) showed, tRNA order, as well as CR organization changed frequently in the evolution of insect mitogenomes.

Evidence for tRNA rearrangement has also been found in other Ephemeroptera families, such as Siphluriscidae [*S. chinensis (Li, Qin & Zhou, 2014)* ], Heptageniidae [*P. youi (Zhang et al., 2008)*, *E. herklotsi (Gao et al., 2018b)*, *Epeorus* sp. JZ-2014 (KJ493406) and *Epeorus* sp. MT-2014 (Tang et al., 2014)], and Baetidae [*A. yixiani* ( GU479735)]. It has also been observed in other insect orders such as Mantodea (Zhang et al., 2018a; Zhang et al., 2018b), Hymenoptera (Cameron et al., 2008) and Lepidoptera (Hu et al., 2010). The translocation of *trnI* to a position between *12S rRNA* and CR also occurred in *Anthonomus* species (Coleoptera: Curculionidae), such as *Anthonomus rectirostris*, *A. rubi*, *A. eugenii* and *A. pomorum* (Van de Vossenberg et al., 2019). However, *Anthonomus* species did not show the inversion of *trnI* that occurs in mayflies. Remarkably, the present data for *T. tumiforceps* and *T. grandipennis* is the first time that three copies of *trnI* inversion and translocation have been reported in Insecta. The rearrangement of *trnI* may be a molecular marker for the family Ephemerellidae, whereas the three copies of *trnI* are probably a genus synapomorphy of the genus *Torleya*. These inferences will require more mitogenomes of Ephemerellidae and *Torleya* species to be fully proven.

Like all other mitogenomes of Ephemeroptera, two rRNAs were detected in these Ephemerellidae species, the *16S rRNA* and *12S rRNA*. The *16S rRNA* was located between *trnL1* (CUN) and *trnV*. Unexpectedly, the *12S rRNA* was located between *trnV* and *trnI* due to the translocation of *trnI*. The sizes of the *16S rRNA* genes in these six mayfly species varied from 1,212 bp (*T. grandipennis*) to 1,230 bp (*Serratella* sp. Yunnan-2018), and the sizes of *12S rRNA* ranged from 769 bp (*T. grandipennis* and *T. tumiforceps*) to 778 bp (*Serratella* sp. Yunnan-2018). Their length fit within the lengths detected in other Ephemeroptera mitogenomes (Tables S3, S4). The mitogenome of *Serratella* sp. Yunnan-2018 had the highest A+T content of the rRNA genes (68.8%) whereas the mitogenome of *T. grandipennis* had the lowest (63.1%). In the six mayfly mitogenomes, the AT bias of both *16S rRNA* and *12S rRNA* was slightly positive (≤0.075), whereas the GC bias was strongly positive (≥0.22), which revealed that the contents of A and G outnumbered T and C nucleotides, respectively (Table 2).

### Non-coding control region

The A+T contents of the CRs of the six mayfly species ranged from 54.5% (*Ephemerella* sp. Yunnan-2018) to 68.5% (*Serratella* sp. Liaoning-2019) (Table 2). The CRs all showed negative AT-skew values and negative GC-skew values. The various sizes of these mitogenomes were largely determined by the amounts and lengths of tandem repeats in the CR. Among insect mitogenomes, a large non-coding region was defined as the A+T region because of the high A+T content, which harbored the origin sites for transcription and replication (Wolstenholme, 1992; Taylor et al., 1993; Yukuhiro et al., 2002). We observed the existence of tandem repeats in the mitochondrial CR in *Ephemerella* sp. Yunnan-2018, *S. zapekinae*, and *Serratella* sp. Liaoning-2019 but not in the other three species analyzed in this study. These consisted of two tandem repeats of a 93 bp sequence and two tandem repeats of a 61 bp as well as one tandem repeat of a 16 bp in *Ephemerella* sp. Yunnan-2018; 2 tandem repeats of a 109 bp sequence as well as one tandem repeat of a 93 bp and three tandem repeats of a 72 bp sequence in *Serratella* sp. Liaoning-2019; and three tandem repeats of a 100 bp sequence, two tandem repeats of a 90 bp sequence and six tandem repeats of a 50 bp sequence in *S. zapekinae* (Fig. S8). It has been proposed that slipped-strand mispairing during mitogenome replication could lead to the appearance of tandem repeat units (Moritz & Brown, 1987). The secondary structure of these repeat units, which could be predicted by Mfold (Zuker, 2003), are shown in Fig. S9. Stable secondary structures were detected in these repeat units except in *Ephemerella* sp. Yunnan-2018, which may make the slipped strand steady or block polymerase for the promotion of replication slippage (Savolainen, Arvestad & Lundeberg, 2000). These structural characteristics may pave the way for revealing the relationship between species evolution and duplicated mitochondrial sequences (Schultheis, Weigt & Hendricks, 2002; Tatarenkov & Avise, 2007; Sammler, Bleidorn & Tiedemann, 2011).

Interestingly, the A+T content of CRs in *Ephemerella* sp. Yunnan-2018, *S. zapekinae* and *Serratella* sp. Yunnan-2018 were obviously lower than the A+T content of corresponding whole mitogenomes, which was also observed in other mayflies (*Paegniodes cupulatus*, *Parafronurus youi*) (Table S6). This phenomenon was also observed in other insects, such as Orthoptera (*Mecopoda niponensis*) and Hemiptera (*Lethocerus deyrollei*, *Aleurocanthus spiniferus*, *Corizus tetraspilus*, *Triatoma dimidiate*, *Hydaropsis longirostris*) (Dotson & Beard, 2001; Hua et al., 2008; Yuan et al., 2015; Yang, Ren & Huang, 2016; Li et al., 2017). The abnormal situation of A+T content of CRs may be affected by A+T content of tandem repeat units. The presence of tandem repeats in the mitochondrial CR has also been detected in many Ephemeroptera species (Zhang et al., 2008; Li, Qin & Zhou, 2014; Zhou et al., 2016; Ye et al., 2018; Cao et al., 2020; Xu et al., 2020). In the CR of *P. youi*, the repeat region included 3 tandem repeats of a 94 bp sequence, which has a lower AT-content of 43.6% (Zhang et al., 2008). Also, the CR of *Paegniodes cupulatus* contained 11 tandem repeats of a 56 bp sequence (Zhou et al., 2016). Moreover, six replicates of a 140 bp sequence and seven partial 76 bp sequences existed in the CR of *S. chinensis*, an observation that is infrequent among the published mitogenomes of mayflies and other insects (Li, Qin & Zhou, 2014). However, the CRs in some species of different families among the Ephemeroptera had no tandem repeat, such as Caenidae [*Caenis* sp*.* YJ-2009 (GQ502451), *Caenis* sp. JYZ-2018 (Cai et al., 2018) and *Caenis* sp. JYZ-2020 (Xu et al., 2020)], Heptageniidae [*E. herklotsi (Gao et al., 2018b)*, *Epeorus* sp. JZ-2014 (KJ493406) and *Epeorus* sp. MT-2014 (Tang et al., 2014)], Siphlonuridae [*Siphlonurus immanis* (FJ606783)] and Isonychiidae [*Isonychia ignota* (HM143892)].

### Intergenic Region

The mitogenomes of *S. zapekinae*, *Serratella* sp. Yunnan-2018 and *Serratella* sp. Liaoning-2019 contained 4, 6, and 6 non-coding intergenic spacer sequences, respectively, with total lengths of 22 bp, 32 bp and 29 bp, whereas *Ephemerella* sp. Yunnan-2018 had 7 non-coding intergenic spacer sequences of 92 bp in total length due to an intergenic spacer of 68 bp between *ND4L* and *trnT*. An intergenic spacer between *trnS2* (UCN) and *ND1* ranging from 17 bp to 19 bp was also detected in these four mitogenomes and is commonly found in Ephemeroptera. In terms of the intergenic spacer between *ND4L* and *trnT* in *Ephemerella* sp. Yunnan-2018, we also detected a similar sequence of 62 bp with a similarity of 72.58% in *Ephemerella* sp. MT-2014 (Tang et al., 2014). Hence, this intergenic spacer is possibly specific to the genus *Ephemerella*. A discussion of the occurrence and function of this intergenic spacer will require mitogenome sequencing of more *Ephemerella* species.

In terms of the mitogenomes of *T. tumiforceps* and *T. grandipennis*, these two mitogenomes both contained 7 intergenic spacers with total lengths of 565 bp and 237 bp. Except for small intergenic spacers (≤12 bp), we observed a large intergenic spacer between *trnS1* (AGN) and *trnE* within the mitogenomes of *T. tumiforceps* and *T. grandipennis*, with lengths of 531 bp and 200 bp, respectively. The large intergenic region in *T. tumiforceps* and *T. grandipennis* had 52.17% and 52% A+T content, respectively, with a strongly positive GC-skew (0.31 and 0.13). The large intergenic region in *T. tumiforceps* and *T. grandipennis* had a similar starting sequence of 20 bp (GGGSCKAAAGGSCCTACCTA) and a conserved sequence of 19 bp (TTTTTAGCGAACTTAGGGG), respectively, with 4 tandem repeats of 87 bp and 3 tandem repeats of 42 bp along with 4 partial sequences, respectively. The repeat unit of *T. tumiforceps* could be folded into three stem-loop secondary structures, whereas *T. grandipennis* folded into two stem-loop secondary structures (Fig. S10). The latter two stem-loops of *T. tumiforceps* had a certain similarity with *T. grandipennis*, which may promote replication slippage and subsequently lead to an increase in duplicate copies (Zhang et al., 2014), acting as a probable substitute replication origin for mtDNA (Crozier & Crozier, 1993; Dotson & Beard, 2001). Thus, it was suggested that this large intergenic region may be the result of the duplication and random loss of the conserved sequence (Fig. S11), similar to the large repetitive sequences between *trnE* and *trnF* in aphids and the intergenic regions in *Parnassius bremeri* reported by Zhang et al. (2014) and Kim et al. (2009), respectively. The large intergenic spacer between *trnS1* (AGN) and *trnE* could be synapomorphic for the genus *Torleya* owing to the fact that sequences located between *trnS1* (AGN) and *trnE* within mitogenomes of other Ephemeroptera were short (<13 bp) and the large intergenic spacer has only been found in *Torleya*. This is the first finding of a large intergenic region in the mitogenomes of mayflies. As a consequence of the non-coding particularity of the intergenic spacer sequence, these species may well have gone through further sequence divergence swiftly (Kim et al., 2009). With the accumulation of more sequence information from *Torleya* species, more instructive and clear conclusions could be given.

The mitogenomes of most insect species are seemingly economical, although certain species contain large intergenic regions (Boore, 1999; Kim et al., 2009; Zhang et al., 2014; Wang et al., 2019). An analysis of intergenic regions in the published mitogenomes of Ephemeroptera revealed the frequent occurrence of quite long intergenic spaces. For example, we observed the same intergenic region of 47 bp between *ND4L* and *trnT* in both Isonychiidae** [*Isonychia kiangsinensis* (Ye et al., 2018) and *I. ignota* (HM143892)], whereas an intergenic spacer between *trnQ* and *trnM* ranging from 26 bp to 53 bp was found in Caenidae [*Caenis* sp*.* YJ-2009 (GQ502451), *Caenis* sp. JYZ-2018 (Cai et al., 2018) and *Caenis* sp. JZY-2020 (Xu et al., 2020)], as well as in Siphlonuridae [*S. immanis* (FJ606783)] and Ephemeridae [*E. orientalis* (EU591678)]. In terms of the mitogenomes of Heptageniidae, we detected an intergenic spacer between *trnA* and *trnR*, ranging from 33 bp to 47 bp (Zhang et al., 2008; Gao et al., 2018b; Song et al., 2019). This intergenic spacer had a high similarity to the A+T region, ranging from 70% to 100%, with lengths ranging from 20 bp to 28 bp. In conclusion, based on intergenic spacers detected in different families and genera of Ephemeroptera, intergenic regions may be specific to family or genus. However, more mitochondrial sequences are needed to support a further discussion of intergenic regions within Ephemeroptera.

### Phylogenetic analyses

**Figure 4:**
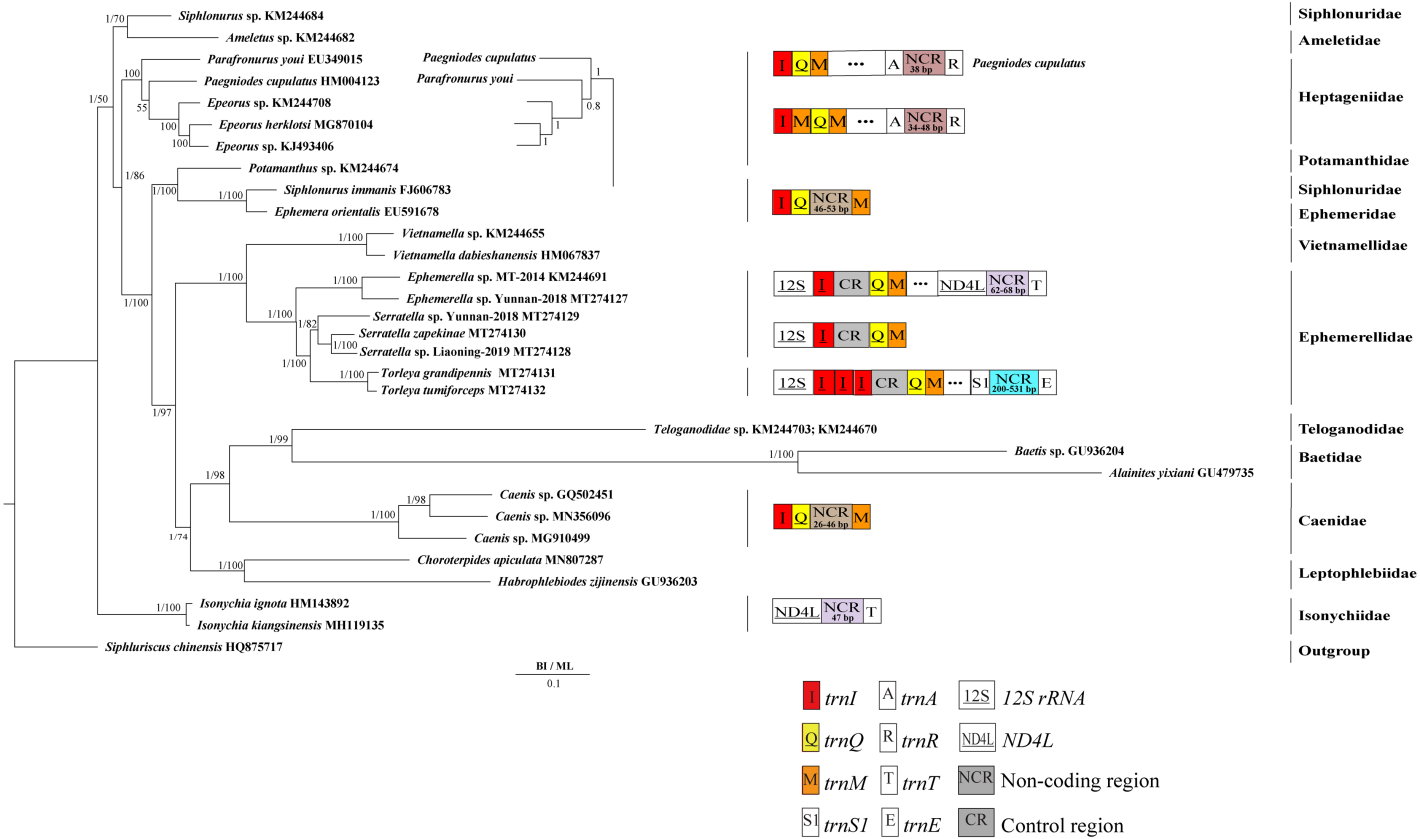
Phylogenetic tree of the relationships among 30 species of Ephemeroptera based on the nucleotide dataset of the 13 mitochondrial protein-coding genes. *Siphluriscus chinensis* was used as the outgroup. The numbers above branches specify posterior probabilities as determined from BI and bootstrap percentages from ML. The GenBank accession numbers of all species are shown in the figure. Gene sizes are not drawn to scale. Box images show gene rearrangements for the six species. Genes encoded by the minority strand are underlined and without underline are encoded by the majority strand. White boxes represent genes with the same relative position as in the ancestral insect arrangement pattern. Different colored boxes represent different genes. The remaining genes and gene orders that were identical to the ancestral insect are left out.

**Figure 5:**
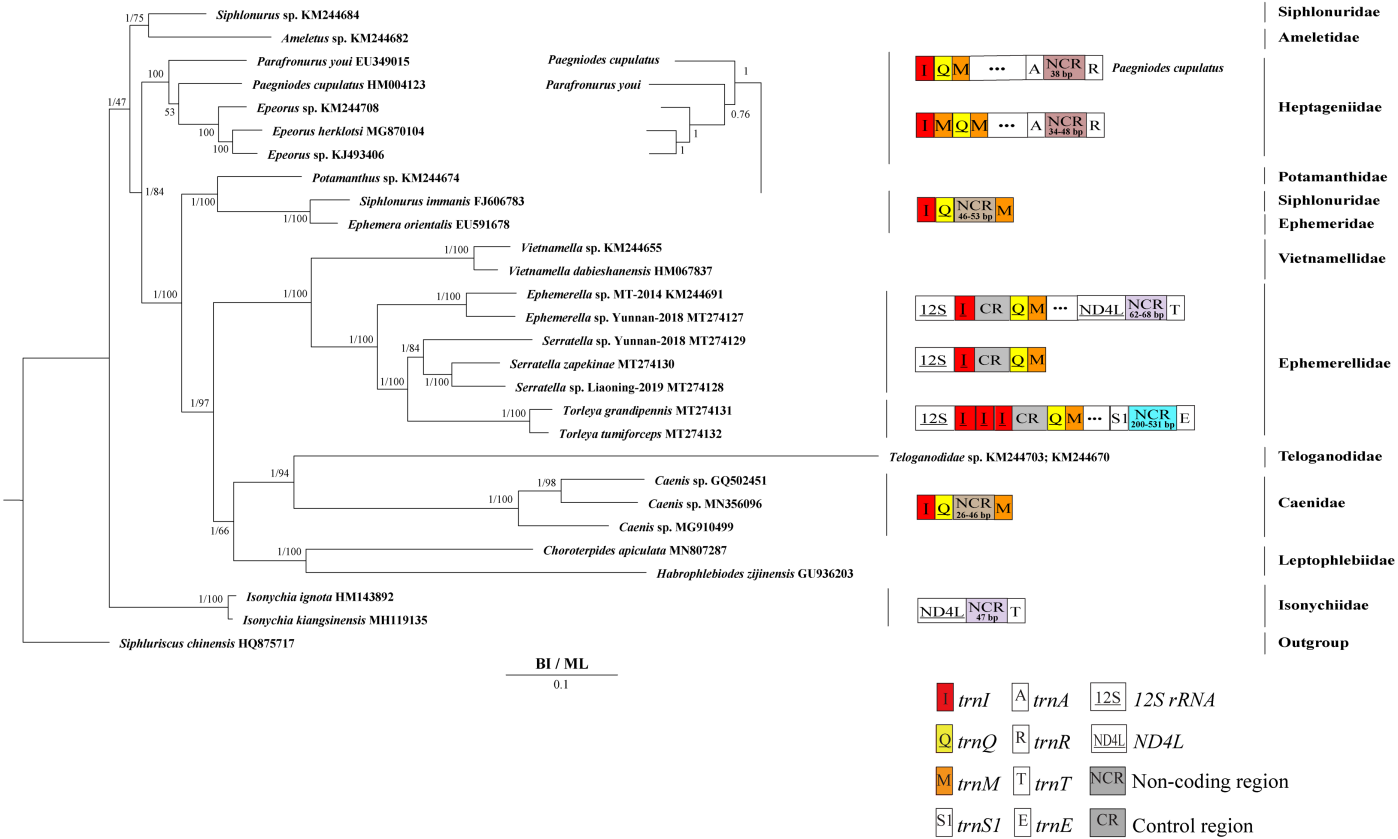
Phylogenetic tree of the relationships among 28 species of Ephemeroptera excluding *Baetis* sp. (GU936204) and *Alainites yixiani* (GU479735) based on the nucleotide dataset of the 13 mitochondrial protein-coding genes. *Siphluriscus chinensis* was used as the outgroup. The numbers above branches specify posterior probabilities as determined from BI and bootstrap percentages from ML. The GenBank accession numbers of all species are shown in the figure. Gene sizes are not drawn to scale. Genes encoded by the minority strand are underlined and without underline are encoded by the majority strand. White boxes represent genes with the same relative position as in the ancestral insect arrangement pattern. Different color boxes represent different genes. The remaining genes and gene orders that were identical to the ancestral insect are left out.

Derived from both BI and ML analyses, the phylogenetic trees showed an identical topology, excluding the internal relationship among Heptageniidae (Figs. 4 and 5). The BI tree showed (*P. cupulatus* + (*P. youi* + (*Epeorus* sp. JZ-2014 + (*Epeorus* sp. MT-2014 + *E. herklotsi*)))), as reported by Cai et al. (2018), Cao et al. (2020) and Xu et al. (2020). On the contrary, the ML tree showed (*P. youi* + (*P. cupulatus* + (*Epeorus* sp. JZ-2014 + (*Epeorus* sp. MT-2014 + *E. herklotsi*)))), as reported by Gao et al. (2018b) and Ye et al. (2018). The difference between the BI and ML analysis was the relationship among *P. youi*, *P. cupulatus* and (*Epeorus* sp. JZ-2014 + (*Epeorus* sp. MT-2014 + *E. herklotsi*)), with low support. On the whole, the monophyly of all Ephemeroptera families was supported except the family Siphluriscidae.

Our findings indicated that Ephemerellidae was the sister clade to Vietnamellidae, but Teloganellidae was the sister clade to Baetidae (Fig. 4), which may be caused by long-branch attraction (LBA). Considering that LBA existed in Baetidae species, we deleted the two species of Baetidae and rebuild the BI and ML phylogenetic trees (Fig. 5). Ephemerellidae was still the sister clade to Vietnamellidae, but Teloganellidae with LBA was a sister clade to Caenidae. Moreover, LBA existed in the family Baetidae, observed in both the BI and ML analysis (Fig. 4). It has been proposed that LBA is caused by a rapid evolutionary rate (Felsenstein, 1978; Brinkmann et al., 2005; Lartillot, Brinkmann & Philippe, 2007; Dabert et al., 2010). Under these circumstances, more slowly evolving species are necessarily needed to eliminate the LBA and reconstruct the phylogenetic relationship within the Ephemeroptera (Kim, 1996; Poe, 2003). The sister-group relationship between Ephemerellidae and Vietnamellidae was supported by current phylogenetic analyses using 13 PCGs of the mayfly mitogenomes (Figs. 4 and 5) as well as the results of many other studies (Cai et al., 2018; Gao et al., 2018b; Ye et al., 2018; Cao et al., 2020; Xu et al., 2020). There has been substantial controversy surrounding the phylogenetic relationships among Ephemerellidae, Vietnamellidae and Teloganellidae. Morphological characteristics have indicated that Teloganellidae and Vietnamellidae have a close phylogenetic relationship. The merged branch of Teloganellidae and Vietnamellidae formed a sister group to Ephemerellidae (McCafferty, 1991; Kluge, 2004). By contrast, via a phylogenetic tree based on the *12S*, *16S*, *18S*, *28S* and *H3* genes, Ephemerellidae was supported as a monophyletic group, and their relationship with Teloganellidae and Vietnamellidae remains problematic (Ogden et al., 2009). In addition, *Ephemerella*, *Serratella* and *Torleya* formed a monophyletic clade within Ephemerellidae. Thus, this phylogenetic tree showed the main topology: ((((*S. zapekinae* + *Serratella* sp. Liaoning-2019) + *Serratella* sp. Yunnan-2018) + (*T. tumiforceps* + *T. grandipennis*)) + (*Ephemerella* sp. Yunnan-2018 + *Ephemerella* sp. MT-2014)). The inversion and translocation of one copy of *trnI* was found in *Ephemerella* and *Serratella*, whereas the inversion and translocation of three copies of *trnI* was found in *Torleya* (Fig. 4). According to the phylogenetic relationship of the three genera in Ephemerellidae ((*Serratella* + *Torleya*) + *Ephemerella*), we can deduce that the inversion and translocation of one copy of *trnI* is characteristic of the ancestral Ephemerellidae. Unfortunately, the presence of only one mitogenome of Teloganellidae restricted a discussion of its monophyly, which requires more species to be added.

## Conclusion

We successfully determined the complete mitogenomes of *Ephemerella* sp. Yunnan-2018, *S. zapekinae*, *Serratella* sp. Yunnan-2018, *Serratella* sp. Liaoning-2019, *T. grandipennis* and *T. tumiforceps*. The mitogenomes of the genera *Ephemerella* and *Serratella* showed similar gene features to *Ephemerella* sp. MT-2014 (KM244691) and had the same inversion and translocation of *trnI*, whereas two extra copies of *trnI* were found in the genus *Torleya*. The translocation and extra copies of *trnI* could be explained by the tandem duplication/random loss model, whereas the inversion of *trnI* could be interpreted by the mitogenome recombination model. The evidence from this study suggests that these specific mitogenome rearrangements may be molecular markers of the family Ephemerellidae, especially the three copies of *trnI* as a synapomorphy for the genus *Torleya*. The occurrences and mechanisms of the large intergenic region between *trnS1* (AGN) and *trnE*, detected in *T. tumiforceps* and *T. grandipennis,* require more mitogenomes of *Torleya* species to be investigated. Phylogenetic analyses based on BI and ML trees both supported the monophyly of Ephemerellidae and Vietnamellidae. Moreover, Ephemerellidae acted as the sister clade to Vietnamellidae whereas Teloganellidae existed in the other clade. Considerable work may be needed to increase the number of mitogenomes to resolve the phylogenetic relationships of Ephemeroptera.

##  Supplemental Information

10.7717/peerj.9740/supp-1 Supplemental Information 1 The data of mitochondrial gnenome of Torleya tumiforceps

10.7717/peerj.9740/supp-2 Supplemental Information 2 The data of mitochondrial gnenome of Torleya grandipennis

10.7717/peerj.9740/supp-3 Supplemental Information 3 The data of mitochondrial gnenome of Serratella sp01

10.7717/peerj.9740/supp-4 Supplemental Information 4 The data of mitochondrial gnenome of Serratella zapekinae

10.7717/peerj.9740/supp-5 Supplemental Information 5 The data of mitochondrial gnenome of Serratella sp02

10.7717/peerj.9740/supp-6 Supplemental Information 6 The data of mitochondrial gnenome of Ephemerella sp

10.7717/peerj.9740/supp-7 Supplemental Information 7 Circular visualization and organization of the complete mitogenomes External genes on the circle are encoded by the positive strand (5’ →3’) and internal genes are encoded by the negative strand (3’ →5’). (A)* Ephemerella* sp. Yunnan-2018, *Serratella zapekinae*,* Serratella* sp. Yunnan-2018. and* Serratella* sp. Liaoning-2019; (B)* Torleya grandipennis* and* Torleya tumiforceps. The figure is written permission from Yu-Rou Cao to publish. She helped drawing under our CC BY 4.0 license.*

10.7717/peerj.9740/supp-8 Supplemental Information 8 Inferred secondary structures of the 22 tRNA genes in *Ephemerella* sp. Yunnan-2018 mitogenome A) *trnI*; (B) *trnQ*; (C) *trnM*; (D) *trnW*; (E) *trnC*; (F) *trnY*; (G) *trnL* (CUN); (H) *trnK*; (I) *trnD*; (J) *trnG*; (K) *trnA*; (L) *trnR*; (M) *trnN*; (N) *trnS* (AGN); (O) *trnE*; (P) *trnF*; (Q) *trnH*; (R) *trnT*; (S) *trnP*; (T) *trnS* (UCN); (U) *trnL* (UUR); (V) *trnV*.

10.7717/peerj.9740/supp-9 Supplemental Information 9 Inferred secondary structures of the 22 tRNA genes in *Serratella zapekinae* mitogenome (A) *trnI*; (B) *trnQ*; (C) *trnM*; (D) *trnW*; (E) *trnC*; (F) *trnY*; (G) *trnL* (CUN); (H) *trnK*; (I) *trnD*; (J) *trnG*; (K) *trnA*; (L) *trnR*; (M) *trnN*; (N) *trnS* (AGN); (O) *trnE*; (P) *trnF*; (Q) *trnH*; (R) *trnT*; (S) *trnP*; (T) *trnS* (UCN); (U) *trnL* (UUR); (V) *trnV*.

10.7717/peerj.9740/supp-10 Supplemental Information 10 Inferred secondary structures of the 22 tRNA genes in *Serratella* sp. Yunnan-2018 mitogenome (A) *trnI*; (B) *trnQ*; (C) *trnM*; (D) *trnW*; (E) *trnC*; (F) *trnY*; (G) *trnL* (CUN); (H) *trnK*; (I) *trnD*; (J) *trnG*; (K) *trnA*; (L) *trnR*; (M) *trnN*; (N) *trnS* (AGN); (O) *trnE*; (P) *trnF*; (Q) *trnH*; (R) *trnT*; (S) *trnP*; (T) *trnS* (UCN); (U) *trnL* (UUR); (V) *trnV*.

10.7717/peerj.9740/supp-11 Supplemental Information 11 Inferred secondary structures of the 22 tRNA genes in *Serratella* sp. Liaoning-2019 mitogenome (A) *trnI*; (B) *trnQ*; (C) *trnM*; (D) *trnW*; (E) *trnC*; (F) *trnY*; (G) *trnL* (CUN); (H) *trnK*; (I) *trnD*; (J) *trnG*; (K) *trnA*; (L) *trnR*; (M) *trnN*; (N) *trnS* (AGN); (O) *trnE*; (P) *trnF*; (Q) *trnH*; (R) *trnT*; (S) *trnP*; (T) *trnS* (UCN); (U) *trnL* (UUR); (V) *trnV*.

10.7717/peerj.9740/supp-12 Supplemental Information 12 Inferred secondary structures of the 24 tRNA genes in *Torleya grandipennis* mitogenome (A) *trnI*; (B) *trnI*; (C) *trnI*; (D)*trnQ*; (E) *trnM*; (F) *trnW*; (G) *trnC*; (H) *trnY*; (I) *trnL* (CUN); (K) *trnK*; (K) *trnD*; (L) *trnG*; (M) *trnA*; (N)*trnR*; (O) *trnN*; (P) *trnS* (AGN); (Q) *trnE*; (R) *trnF*; (S) *trnH*; (T) *trnT*; (U) *trnP*; (V) *trnS* (UCN); (W) *trnL* (UUR); (X) *trnV*.

10.7717/peerj.9740/supp-13 Supplemental Information 13 Inferred secondary structures of the 24 tRNA genes in *Torleya tumiforceps* mitogenome (A) *trnI*; (B) *trnI*; (C) *trnI*; (D)*trnQ*; (E) *trnM*; (F) *trnW*; (G) *trnC*; (H) *trnY*; (I) *trnL* (CUN); (K) *trnK*; (K) *trnD*; (L) *trnG*; (M) *trnA*; (N)*trnR*; (O) *trnN*; (P) *trnS* (AGN); (Q) *trnE*; (R) *trnF*; (S) *trnH*; (T) *trnT*; (U) *trnP*; (V) *trnS* (UCN); (W) *trnL* (UUR); (X) *trnV*.

10.7717/peerj.9740/supp-14 Supplemental Information 14 Organizations of the repeat regions in CR of Ephemerella sp. Yunnan-2018, Serratella sp. Liaoning-2019 and S. zapekinae

10.7717/peerj.9740/supp-15 Supplemental Information 15 The possible secondary structure of the tandem repeat in the control regions. (A) the repeat unit (90 bp) in *Serratella zapekinae*;(B) the repeat unit (100 bp) in* Serratella zapekinae*;(C) the repeat unit (109 bp) in* Serratella* sp. Liaoning-2019; (D) the repeat unit (72 bp) in* Serratella* sp. Liaoning-2019.

10.7717/peerj.9740/supp-16 Supplemental Information 16 The possible secondary structure of the tandem repeat in the intergenic region between *trnS1* (AGN) and *trnE* of *Torleya tumiforceps* and *T. grandipennis*. (A) the repeat unit (87 bp) in *T. tumiforceps*; (B) the repeat unit (42 bp) in *T. grandipennis*.

10.7717/peerj.9740/supp-17 Supplemental Information 17 Putative mechanisms for formation of the large non-coding region (NCR) that exists in *Torleya grandipennis* and *Torleya tumiforceps* The duplication/random loss model can explain the NCR between *trnS1* (AGN) and *trnE*. The CS indicates the 19 bp conserved sequence TTTTTAGCGAACTTAGGGG. Gene sizes are not drawn to scale. Genes located on the majority strand are shown along the top of the boxes whereas genes located on the minority strand are shown on the bottom. White boxes represent genes with the same relative position as in the ancestral insect arrangement pattern. Grey boxes represent non-coding regions. The remaining genes and gene orders that were identical to the ancestral insect are left out.

10.7717/peerj.9740/supp-18 Supplemental Information 18 Universal primers and specific primers used to amplify the mitogenomes of six mayfly mitochondrial genomes

10.7717/peerj.9740/supp-19 Supplemental Information 19 The partition schemes and best-fitting models selected of 13 protein-coding genes

10.7717/peerj.9740/supp-20 Supplemental Information 20 Location of features in the mtDNA of *Ephemerella* sp. Yunnan-2018, *Serratella zapekinae*, *Serratella* sp. Yunnan-2018 and *Serratella* sp. Liaoning-2019 Orange shading represents *Ephemerella* sp. Yunnan-2018. Blue shading represents *Serratella zapekinae*. Yellow shading represents *Serratella* sp. Yunnan-2018. Green shading represents *Serratella* sp. Liaoning-2019.

10.7717/peerj.9740/supp-21 Supplemental Information 21 Location of features in the mtDNA of *Torleya grandipennis* and *Torleya tumiforceps*. Pink shading represents *Torleya grandipennis*. Purple shading represents *Torleya tumiforceps.*

10.7717/peerj.9740/supp-22 Supplemental Information 22 The codon number and relative synonymous codon usage in mitochondrial protein-coding genes

10.7717/peerj.9740/supp-23 Supplemental Information 23 Ephemeroptera species with the A+T content of the control region and whole mitogenome including six species involved in this study

## Additional Information and Declarations

### Field Study Permissions

The following information was supplied relating to field study approvals (i.e., approving body and any reference numbers): Field experiments were approved by the Research Council of Zhejiang Normal University.

### DNA Deposition

The following information was supplied regarding the deposition of DNA sequences: The six mitogenomes are available at GenBank: MT274127–MT274132.

### Data Availability

The following information was supplied regarding data availability: The raw mitochondrial genomes are available in the Supplementary Files.

## References

[ref-1] Anderson S, Bankier AT, Barrell BG, De Bruijn MHL, Coulson AR, Drouin J, Eperon IC, Nierlich DP, Roe BA, Sanger F, Schreier PH, Smith AJH, Staden R, Young IG (1981). Sequence and organization of the human mitochondrial genome. Nature.

[ref-2] Arndt A, Smith MJ (1998). Mitochondrial gene rearrangement in the sea cucumber genus *Cucumaria*. Molecular Biology and Evolution.

[ref-3] Benson G (1999). Tandem repeats finder: a program to analyze DNA sequences. Nucleic Acids Research.

[ref-4] Bernt M, Donath A, Jühling F, Externbrink F, Florentz C, Fritzsch G, Pütz J, Middendorf M, Stadler PF (2013). MITOS: improved de novo metazoan mitochondrial genome annotation. Molecular Phylogenetics and Evolution.

[ref-5] Boore JL (1999). Animal mitochondrial genomes. Nucleic Acids Research.

[ref-6] Boore JL (2006). The use of genome-level characters for phylogenetic reconstruction. Trends in Ecology & Evolution.

[ref-7] Brinkmann H, Van der Giezen M, Zhou Y, De Raucourt GP, Philippe H (2005). An empirical assessment of long-branch attraction artefacts in deep eukaryotic phylogenomics. Systematic Biology.

[ref-8] Burland TG, Misener S, Krawetz SA (1999). DNASTAR’s Lasergene sequence analysis software. Bioinformatics methods and protocols.

[ref-9] Cai YY, Gao YJ, Zhang LP, Yu DN, Storey KB, Zhang JY (2018). The mitochondrial genome of *Caenis* sp. (Ephemeroptera: Caenidae) and the phylogeny of Ephemeroptera in Pterygota. Mitochondrial DNA Part B.

[ref-10] Cameron SL (2014a). Insect mitochondrial genomics: implications for evolution and phylogeny. Annual Review of Entomology.

[ref-11] Cameron SL (2014b). How to sequence and annotate insect mitochondrial genomes for systematic and comparative genomics research. Systematic Entomology.

[ref-12] Cameron SL, Dowton M, Castro LR, Ruberu K, Whiting MF, Austin AD, Diement K, Stevens J (2008). Mitochondrial genome organization and phylogeny of two vespid wasps. Genome.

[ref-13] Cao SS, Xu XD, Jia YY, Guan JY, Storey KB, Yu DN, Zhang JY (2020). The complete mitochondrial genome of *Choroterpides apiculata* (Ephemeroptera: Leptophlebiidae) and its phylogenetic relationships. Mitochondrial DNA Part B.

[ref-14] Carapelli A, Li P, Nardi F, Wath Evander, Frati F (2007). Phylogenetic analysis of mitochondrial protein coding genes confirms the reciprocal paraphyly of Hexapoda and Crustacea. BMC Evolutionary Biology.

[ref-15] Castresana J (2000). Selection of conserved blocks from multiple alignments for their use in phylogenetic analysis. Molecular Biology and Evolution.

[ref-16] Cheng XF, Zhang LP, Yu DN, Storey KB, Zhang JY (2016). The complete mitochondrial genomes of four cockroaches (Insecta: Blattodea) and phylogenetic analyses within cockroaches. Gene.

[ref-17] Conant GC, Wolfe KH (2008). GenomeVx: simple web-based creation of editable circular chromosome maps. Bioinformatics.

[ref-18] Crozier RH, Crozier YC (1993). The mitochondrial genome of the honeybee *Apis mellifera*: complete sequence and genome organization. Genetics.

[ref-19] Dabert M, Witalinski W, Kazmierski A, Olszanowski Z, Dabert J (2010). Molecular phylogeny of acariform mites (Acari, Arachnida): strong conflict between phylogenetic signal and long-branch attraction artifacts. Molecular Phylogenetics and Evolution.

[ref-20] Dickey AM, Kumar V, Morgan JK, Jara-Cavieres A, Shatters RG, McKenzie CL, Osborne LS (2015). A novel mitochondrial genome architecture in thrips (Insecta: Thysanoptera): extreme size asymmetry among chromosomes and possible recent control region duplication. BMC Genomics.

[ref-21] Dotson EM, Beard CB (2001). Sequence and organization of the mitochondrial genome of the Chagas disease vector, *Triatoma dimidiata*. Insect Molecular Biology.

[ref-22] Dowton M, Cameron SL, Dowavic JI, Austin AD, Whiting MF (2009). Characterization of 67 mitochondrial tRNA gene rearrangements in the Hymenoptera suggests that mitochondrial tRNA gene position is selectively neutral. Molecular Biology and Evolution.

[ref-23] Felsenstein J (1978). Cases in which parsimony or compatibility methods will be positively misleading. Systematic Zoology.

[ref-24] Foster PG, Jermiin LS, Hickey DA (1997). Nucleotide composition bias affects amino acid content in proteins coded by animal mitochondria. Journal of Molecular Evolution.

[ref-25] Gao XY, Cai YY, Yu DN, Storey KB, Zhang JY (2018a). Characteristics of the complete mitochondrial genome of *Suhpalacsa longialata* (Neuroptera, Ascalaphidae) and its phylogenetic implications. PeerJ.

[ref-26] Gao XY, Zhang SS, Zhang LP, Yu DN, Zhang JY, Cheng HY (2018b). The complete mitochondrial genome of *Epeorus herklotsi* (Ephemeroptera: Heptageniidae) and its phylogeny. Mitochondrial DNA Part B.

[ref-27] Hu J, Zhang DX, Hao JS, Huang DY, Cameron S, Zhu CD (2010). The complete mitochondrial genome of the yellow coaster, Acraea issoria (Lepidoptera: Nymphalidae: Heliconiinae: Acraeini): sequence gene organization and a unique tRNA translocation event. Molecular Biology Reports.

[ref-28] Hua JM, Li M, Dong PZ, Cui Y, Xie Q, Bu WJ (2008). Comparative and phylogenomic studies on the mitochondrial genomes of Pentatomomorpha (Insecta: Hemiptera: Heteroptera). BMC Genomics.

[ref-29] Jacobus LM, Macadam CR, Sartori M (2019). Mayflies (Ephemeroptera) and their contributions to ecosystem services. Insects.

[ref-30] Kearse M, Moir R, Wilson A, Stones-Havas S, Cheung M, Sturrock S, Buxton S, Cooper A, Markowitz S, Duran C, Thierer T, Ashton B, Meintjes P, Drummond A (2012). Geneious basic: an integrated and extendable desktop software platform for the organization and analysis of sequence data. Bioinformatics.

[ref-31] Kerpedjiev P, Hammer S, Hofacker IL (2015). Forna (force-directed RNA): simple and effective online RNA secondary structure diagrams. Bioinformatics.

[ref-32] Kim J (1996). General inconsistency conditions for maximum parsimony: effects of branch lengths and increasing numbers of taxa. Systematic Biology.

[ref-33] Kim MI, Baek JY, Kim MJ, Jeong HC, Kim KG, Bae CH, Han YS, Jin BR, Kim I (2009). Complete nucleotide sequence and organization of the mitogenome of the red-spotted apollo butterfly, *Parnassius bremeri* (Lepidoptera: Papilionidae) and comparison with other lepidopteran insects. Molecules and Cells.

[ref-34] Kluge NJ (2004). The phylogenetic system of ephemeroptera (the first experience in consistently non-ranking taxonomy).

[ref-35] Kumar S, Stecher G, Tamura K (2016). MEGA7: molecular evolutionary genetics analysis version 7.0 for bigger datasets. Molecular Biology and Evolution.

[ref-36] Lalitha S (2000). Primer premier 5. Biotech Software and Internet Report.

[ref-37] Lanfear R, Calcott B, Ho SYW, Guindon S (2012). PartitionFinder: combined selection of partitioning schemes and substitution models for phylogenetic analyses. Molecular Biology and Evolution.

[ref-38] Lartillot N, Brinkmann H, Philippe H (2007). Suppression of long-branch attraction artefacts in the animal phylogeny using a site-heterogeneous model. BMC Evolutionary Biology.

[ref-39] Leavitt JR, Hiatt KD, Whiting MF, Song H (2013). Searching for the optimal data partitioning strategy in mitochondrial phylogenomics: A phylogeny of Acridoidea (Insecta: Orthoptera: Caelifera) as a case study. Molecular Phylogenetics and Evolution.

[ref-40] Li H, Leavengood Jr JM, Chapman EG, Burkhardt D, Song F, Jiang P, Liu J, Zhou X, Cai W (2017). Mitochondrial phylogenomics of Hemiptera reveals adaptive innovations driving the diversification of true bugs. Proceedings of the Royal Society B: Biological Sciences.

[ref-41] Li D, Qin JC, Zhou CF (2014). The phylogeny of Ephemeroptera in Pterygota revealed by the mitochondrial genome of *Siphluriscus chinensis* (Hexapoda: Insecta). Gene.

[ref-42] Lunt DH, Hyman BC (1997). Animal mitochondrial DNA recombination. Nature.

[ref-43] Ma Y, He K, Yu PP, Yu DN, Cheng XF, Zhang JY (2015). The complete mitochondrial genomes of three bristletails (Insecta: Archaeognatha): the paraphyly of Machilidae and insights into Archaeognathan phylogeny. PLOS ONE.

[ref-44] Macey JR, Larson A, Ananjeva NB, Fang Z, Papenfuss TJ (1997). Two novel gene orders and the role of light-strand replication in rearrangement of the vertebrate mitochondrial genome. Molecular Biology and Evolution.

[ref-45] McCafferty WP (1991). Toward a phylogenetic classification of the Ephemeroptera (Insecta): a commentary on systematics. Annals of the Entomological Society of America.

[ref-46] McCafferty WP, Edmunds Jr GF (1979). The higher classification of the Ephemeroptera and its evolutionary basis. Annals of the Entomological Society of America.

[ref-47] McClain WH (2006). Surprising contribution to aminoacylation and translation of non-Watson-Crick pairs in tRNA. Proceedings of the National Academy of Sciences of the United States of America.

[ref-48] Min XJ, Hickey DA (2007). DNA asymmetric strand bias affects the amino acid composition of mitochondrial proteins. DNA Research.

[ref-49] Moritz C, Brown WM (1987). Tandem duplications in animal mitochondrial DNAs: variation in incidence and gene content among lizards. Proceedings of the National Academy of Sciences of the United States of America.

[ref-50] Moritz C, Dowling TE, Brown WM (1987). Evolution of animal mitochondrial DNA: relevance for population biology and systematics. Annual Review of Ecology and Systematics.

[ref-51] Ogden TH, Gattolliat JL, Sartori M, Staniczek AH, Sold NT, Whiting MF (2009). Towards a new paradigm in mayfly phylogeny (Ephemeroptera): combined analysis of morphological and molecular data. Systematic Entomology.

[ref-52] Ogden TH, Whiting MF (2005). Phylogeny of Ephemeroptera (mayflies) based on molecular evidence. Molecular Phylogenetics and Evolution.

[ref-53] Ojala D, Montoya J, Attardi G (1981). tRNA punctuation model of RNA processing in human mitochondria. Nature.

[ref-54] Oliveira DCSG, Raychoudhury R, Lavrov DV, Werren JH (2008). Rapidly evolving mitochondrial genome and directional selection in mitochondrial genes in the parasitic wasp *Nasonia* (Hymenoptera: Pteromalidae). Molecular Biology and Evolution.

[ref-55] Perna NT, Kocher TD (1995). Patterns of nucleotide composition at fourfold degenerate sites of animal mitochondrial genomes. Journal of Molecular Evolution.

[ref-56] Poe S (2003). Evaluation of the strategy of long-branch subdivision to improve the accuracy of phylogenetic methods. Systematic Biology.

[ref-57] Powell JR, Moriyama EN (1997). Evolution of codon usage bias in *Drosophila*. Proceedings of the National Academy of Sciences of the United States of America.

[ref-58] Rao YS, Wu GZ, Wang ZF, Chai XW, Nie QH, Zhang XQ (2011). Mutation bias is the driving force of codon usage in the *Gallus gallus* genome. DNA Research.

[ref-59] Ronquist F, Teslenko M, Van der Mark P, Ayres DL, Darling A, Höhna S, Larget B, Liu L, Suchard MA, Huelsenbeck JP (2012). MrBayes 3.2: efficient Bayesian phylogenetic inference and model choice across a large model space. Systematic Biology.

[ref-60] Salles FF, nguez EDomí, Molineri C, Boldrini R, Nieto C, Dias LG, Hamada N Thorp JH, Rogers DC (2018). Order Ephemeroptera. Thorp and Covich’s freshwater invertebrates.

[ref-61] Sammler S, Bleidorn C, Tiedemann R (2011). Full mitochondrial genome sequences of two endemic Philippine hornbill species (Aves: Bucerotidae) provide evidence for pervasive mitochondrial DNA recombination. BMC Genomics.

[ref-62] Savolainen P, Arvestad L, Lundeberg J (2000). mtDNA tandem repeats in domestic dogs and wolves: mutation mechanism studied by analysis of the sequence of imperfect repeats. Molecular Biology and Evolution.

[ref-63] Schultheis AS, Weigt LA, Hendricks AC (2002). Arrangement and structural conservation of the mitochondrial control region of two species of Plecoptera: utility of tandem repeat-containing regions in studies of population genetics and evolutionary history. Insect Molecular Biology.

[ref-64] Simon C, Buckley TR, Frati F, Stewart JB, Beckenbach AT (2006). Incorporating molecular evolution into phylogenetic analysis, and a new compilation of conserved polymerase chain reaction primers for animal mitochondrial DNA. Annual Review of Ecology, Evolution, and Systematics.

[ref-65] Simon C, Frati F, Beckenbach A, Crespi B, Liu H, Flook P (1994). Evolution, weighting, and phylogenetic utility of mitochondrial gene sequences and a compilation of conserved polymerase chain reaction primers. Annals of the Entomological Society of America.

[ref-66] Song N, Li XX, Yin XM, Li XH, Yin J, Pan PL (2019). The mitochondrial genomes of palaeopteran insects and insights into the early insect relationships. Scientific Reports.

[ref-67] Stamatakis A (2014). RAxML version 8: a tool for phylogenetic analysis and post-analysis of large phylogenies. Bioinformatics.

[ref-68] Tang M, Tan MH, Meng GL, Yang SZ, Su X, Liu SL, Song WH, Li YY, Wu Q, Zhang AB, Zhou X (2014). Multiplex sequencing of pooled mitochondrial genomes—a crucial step toward biodiversity analysis using mito-metagenomics. Nucleic Acids Research.

[ref-69] Tatarenkov A, Avise J (2007). Rapid concerted evolution in animal mitochondrial DNA. Proceedings Biological Sciences/The Royal Society.

[ref-70] Taylor MF, McKechnie SW, Pierce N, Kreitman M (1993). The lepidopteran mitochondrial control region: structure and evolution. Molecular Biology and Evolution.

[ref-71] Thompson JD, Gibson TJ, Plewniak F, Jeanmougin F, Higgins DG (1997). The CLUSTAL_X windows interface: flexible strategies for multiple sequence alignment aided by quality analysis tools. Nucleic Acids Research.

[ref-72] Varani G, McClain WH (2000). The G x U wobble base pair. A fundamental building block of RNA structure crucial to RNA function in diverse biological systems. EMBO reports.

[ref-73] Van de Vossenberg BTLH, Warbroek T, Ingerson-Mahar J, Waalwijk C, Gouw L, Eichinger B, Loomans A (2019). Tracking outbreak populations of the pepper weevil *Anthonomus eugenii* (Coleoptera; Curculionidae) using complete mitochondrial genomes. PLOS ONE.

[ref-74] Wan XL, Kim M, Kim MJ, Kim I (2012). Complete mitochondrial genome of the free-living earwig, *Challia fletcheri* (Dermaptera: Pygidicranidae) and phylogeny of Polyneoptera. PLOS ONE.

[ref-75] Wang J, Dai XY, Xu XD, Zhang ZY, Yu DN, Storey KB, Zhang JY (2019). The complete mitochondrial genomes of five longicorn beetles (Coleoptera: Cerambycidae) and phylogenetic relationships within Cerambycidae. PeerJ.

[ref-76] Wei SJ, Li Q, Achterberg Kvan, Chen XX (2014). Two mitochondrial genomes from the families Bethylidae and Mutillidae: independent rearrangement of protein-coding genes and higher-level phylogeny of the Hymenoptera. Molecular Phylogenetics and Evolution.

[ref-77] Wei SJ, Shi M, Chen XX, Sharkey MJ, Achterberg Cvan, Ye GY, He JH (2010). New views on strand asymmetry in insect mitochondrial genomes. PLOS ONE.

[ref-78] Wolstenholme DR (1992). Animal mitochondrial DNA: structure and evolution. International Review of Cytology.

[ref-79] Xu XD, Jia YY, Dai XY, Ma JL, Storey KB, Zhang JY, Yu DN (2020). The mitochondrial genome of *Caenis* sp. (Ephemeroptera: Caenidae) from Fujian and the phylogeny of Caenidae within Ephemeroptera. Mitochondrial DNA Part B.

[ref-80] Yang J, Ren Q, Huang Y (2016). Complete mitochondrial genomes of three crickets (Orthoptera: Gryllidae) and comparative analyses within Ensifera mitogenomes. Zootaxa.

[ref-81] Ye QM, Zhang SS, Cai YY, Storey KB, Yu DN, Zhang JY (2018). The complete mitochondrial genome of *Isonychia kiangsinensis* (Ephemeroptera: Isonychiidae). Mitochondrial DNA Part B.

[ref-82] Yuan ML, Zhang QL, Guo ZL, Wang J, Shen YY (2015). The complete mitochondrial genome of *Corizus tetraspilus* (Hemiptera: Rhopalidae) and phylogenetic analysis of pentatomomorpha. PLOS ONE.

[ref-83] Yukuhiro K, Sezutsu H, Itoh M, Shimizu K, Banno Y (2002). Significant levels of sequence divergence and gene rearrangements have occurred between the mitochondrial genomes of the wild mulberry silkmoth, *Bombyx mandarina*, and its close relative, the domesticated silkmoth, *Bombyx mori*. Molecular Biology and Evolution.

[ref-84] Zhang LP, Cai YY, Yu DN, Storey KB, Zhang JY (2018a). Gene characteristics of the complete mitochondrial genomes of *Paratoxodera polyacantha* and *Toxodera hauseri* (Mantodea: Toxoderidae). PeerJ.

[ref-85] Zhang DX, Hewitt GM (1997). Insect mitochondrial control region: a review of its structure, evolution and usefulness in evolutionary studies. Biochemical Systematics and Ecology.

[ref-86] Zhang D, Gao F, Jakovlic I, Zou H, Zhang J, Li WX, Wang GT (2020). PhyloSuite: an integrated and scalable desktop platform for streamlined molecular sequence data management and evolutionary phylogenetics studies. Molecular Ecology Resources.

[ref-87] Zhang B, Ma C, Edwards O, Fuller S, Kang L (2014). The mitochondrial genome of the Russian wheat aphid *Diuraphis noxia*: large repetitive sequences between *trnE* and *trnF* in aphids. Gene.

[ref-88] Zhang LP, Yu DN, Storey KB, Cheng HY, Zhang JY (2018b). Higher tRNA gene duplication in mitogenomes of praying mantises (Dictyoptera, Mantodea) and the phylogeny within Mantodea. International Journal of Biological Macromolecules.

[ref-89] Zhang JY, Zhou CF, Gai YH, Song DX, Zhou KY (2008). The complete mitochondrial genome of *Parafronurus youi* (insecta: Ephemeroptera) and phylogenetic position of the Ephemeroptera. Gene.

[ref-90] Zhou D, Wang YY, Sun JZ, Han YK, Zhou CF (2016). The complete mitochondrial genome of *Paegniodes cupulatus* (Ephemeroptera: Heptageniidae). Mitochondrial DNA Part A.

[ref-91] Zuker M (2003). Mfold web server for nucleic acid folding and hybridization prediction. Nucleic Acids Research.

