# Supplementary material for: Six complete mitochondrial genomes of mayflies from three genera of Ephemerellidae (Insecta: Ephemeroptera) with inversion and translocation of trnI rearrangement and their phylogenetic relationships
Source: PeerJ. 2020 Aug 19;8:e9740. doi: 10.7717/peerj.9740 (PMC7443110; doi:10.7717/peerj.9740)

A. the repeat unit (90 bp) in *Serratella zapekinae*

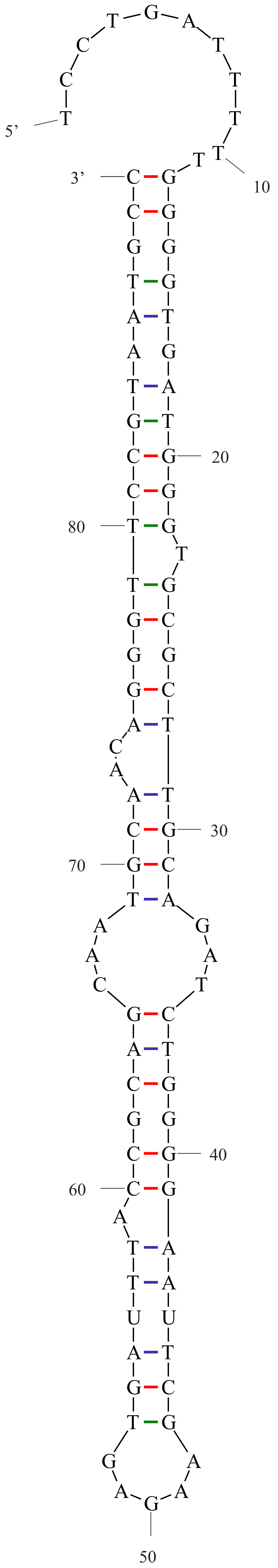

B. the repeat unit (100 bp) in *Serratella zapekinae*

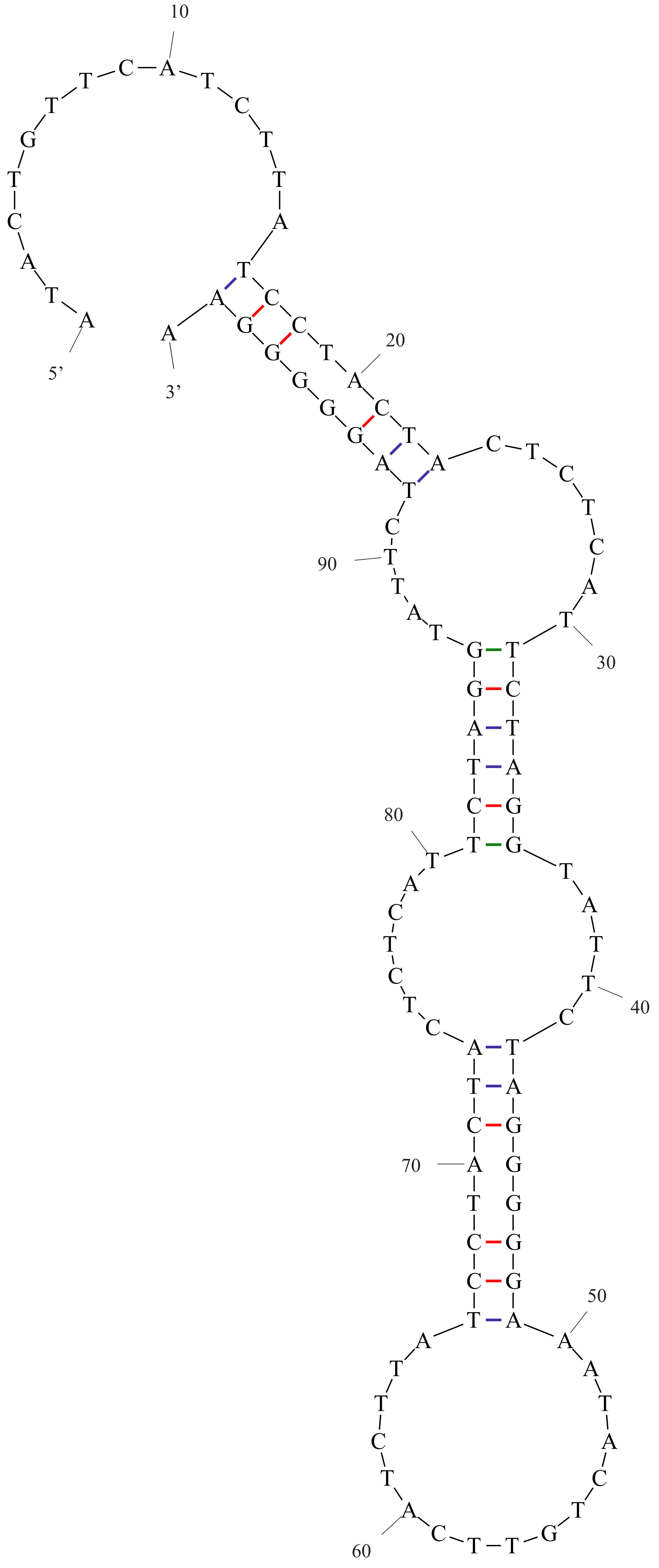

C. the repeat unit (109 bp) in *Serratella* sp. Liaoning-2019

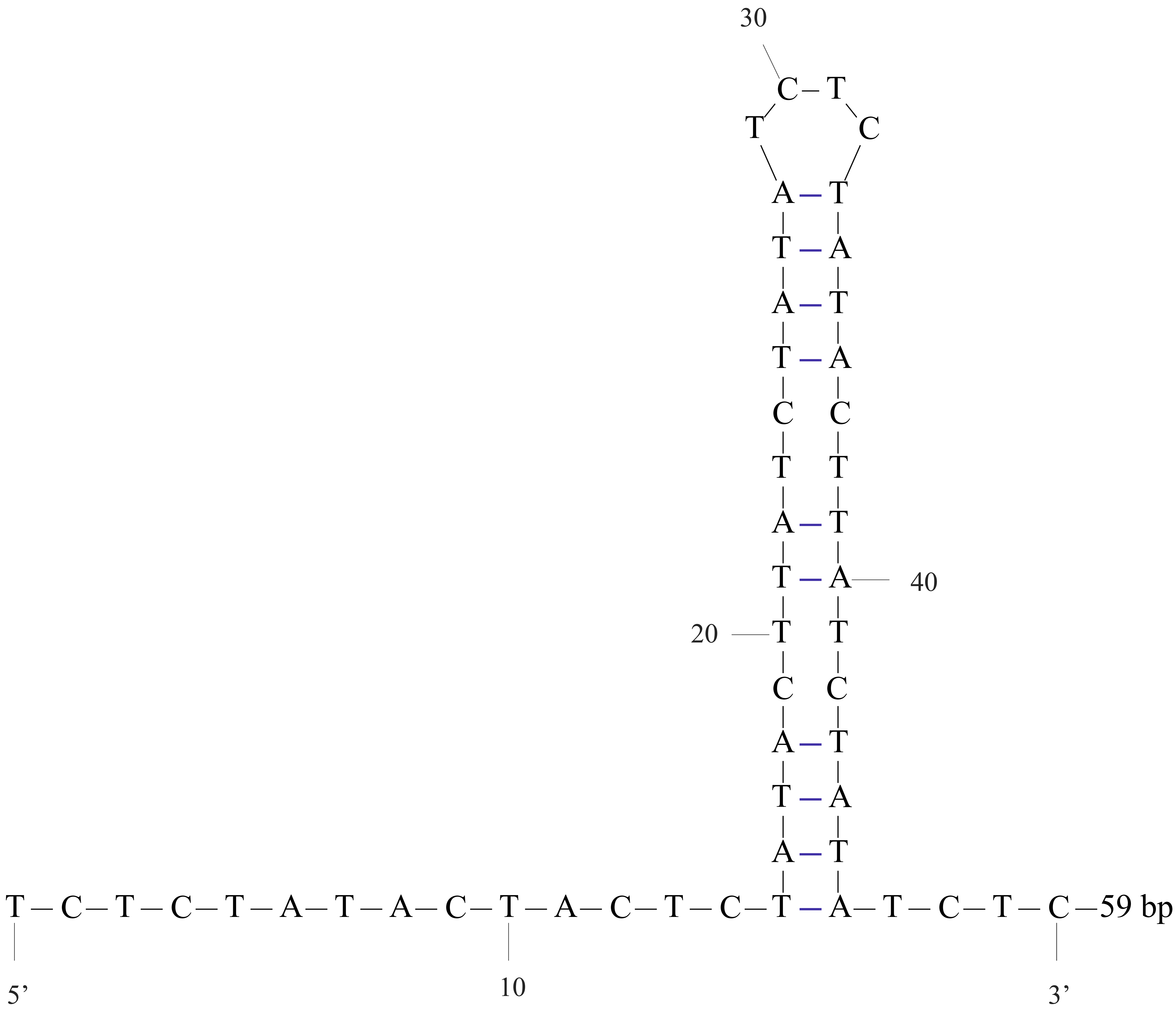

D. the repeat unit (72 bp) in *Serratella* sp. Liaoning-2019

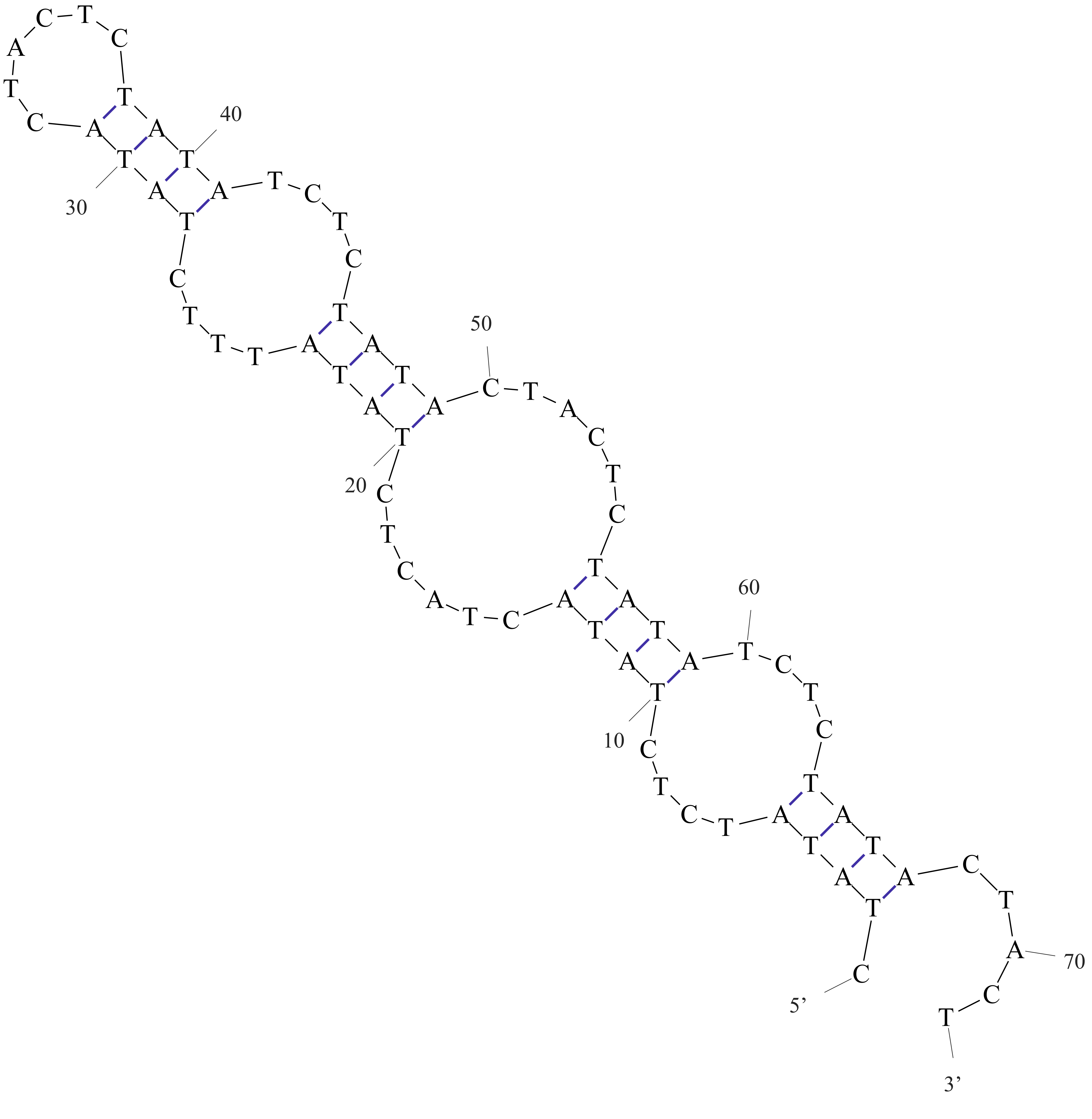

Supplement: Supplemental Information 15 — (A) the repeat unit (90 bp) in Serratella zapekinae;(B) the repeat unit (100 bp) in Serratella zapekinae;(C) the repeat unit (109 bp) in Serratella sp. Liaoning-2019; (D) the repeat unit (72 bp) in Serratella sp. Liaoning-2019. [file peerj-08-9740-s015.pdf]
