# Supplementary figures and images for: Six complete mitochondrial genomes of mayflies from three genera of Ephemerellidae (Insecta: Ephemeroptera) with inversion and translocation of trnI rearrangement and their phylogenetic relationships
Source: PeerJ. 2020 Aug 19;8:e9740. doi: 10.7717/peerj.9740 (PMC7443110; doi:10.7717/peerj.9740)

### A. *Torleya tumiforceps*

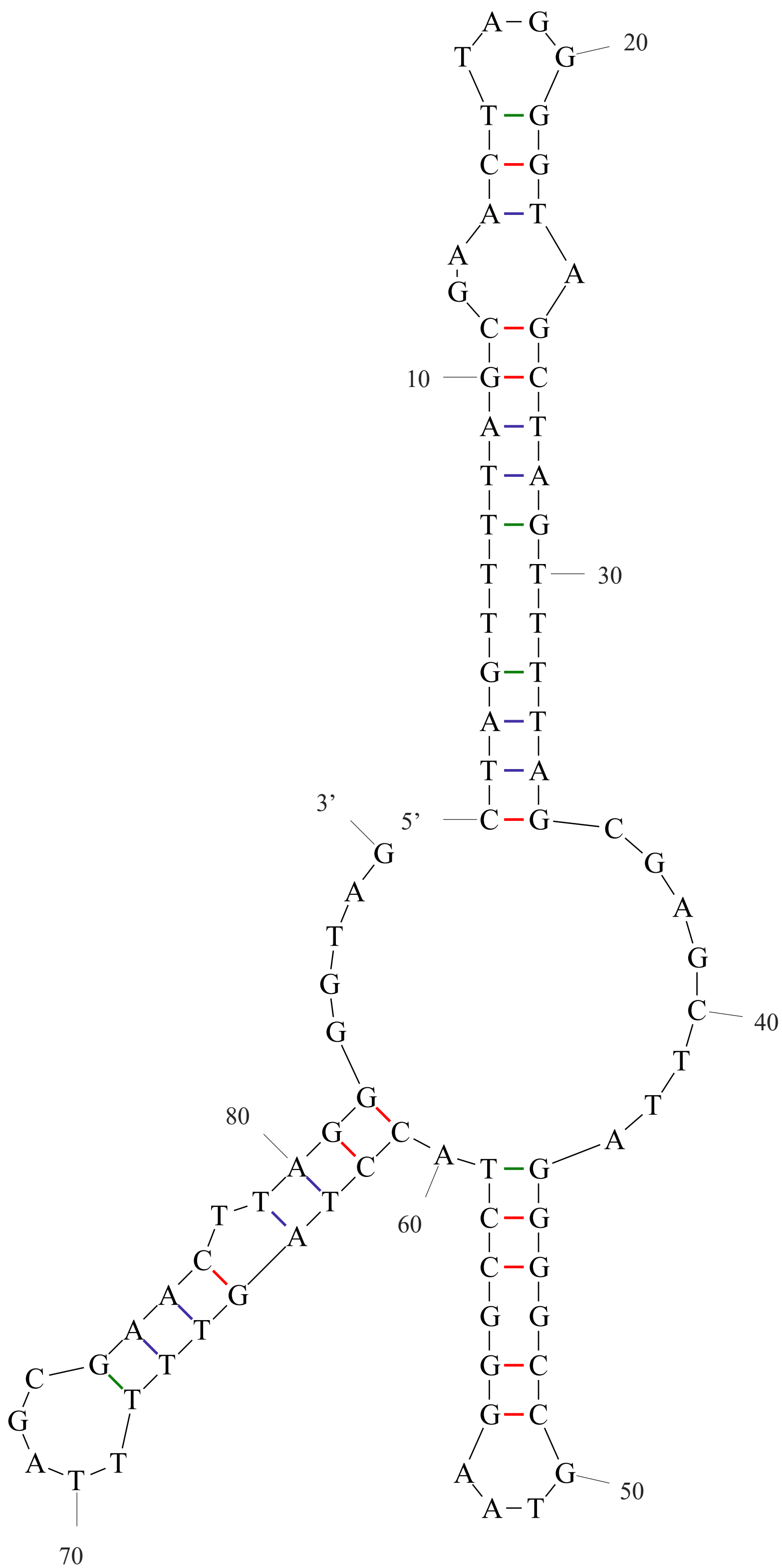

**B. *Torleya grandipennis***

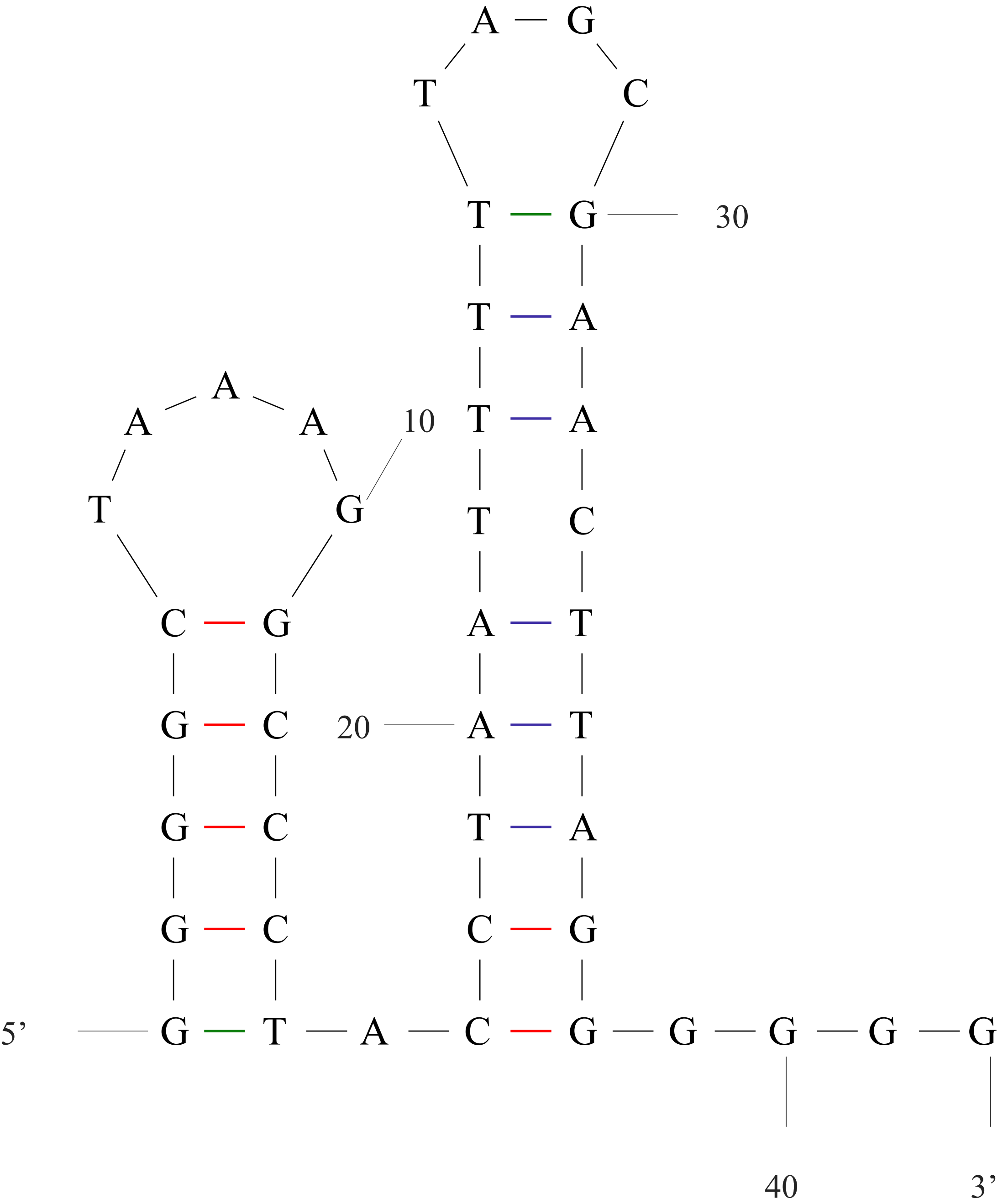

Supplement: Supplemental Information 16 — (A) the repeat unit (87 bp) in T. tumiforceps; (B) the repeat unit (42 bp) in T. grandipennis. [file peerj-08-9740-s016.pdf]

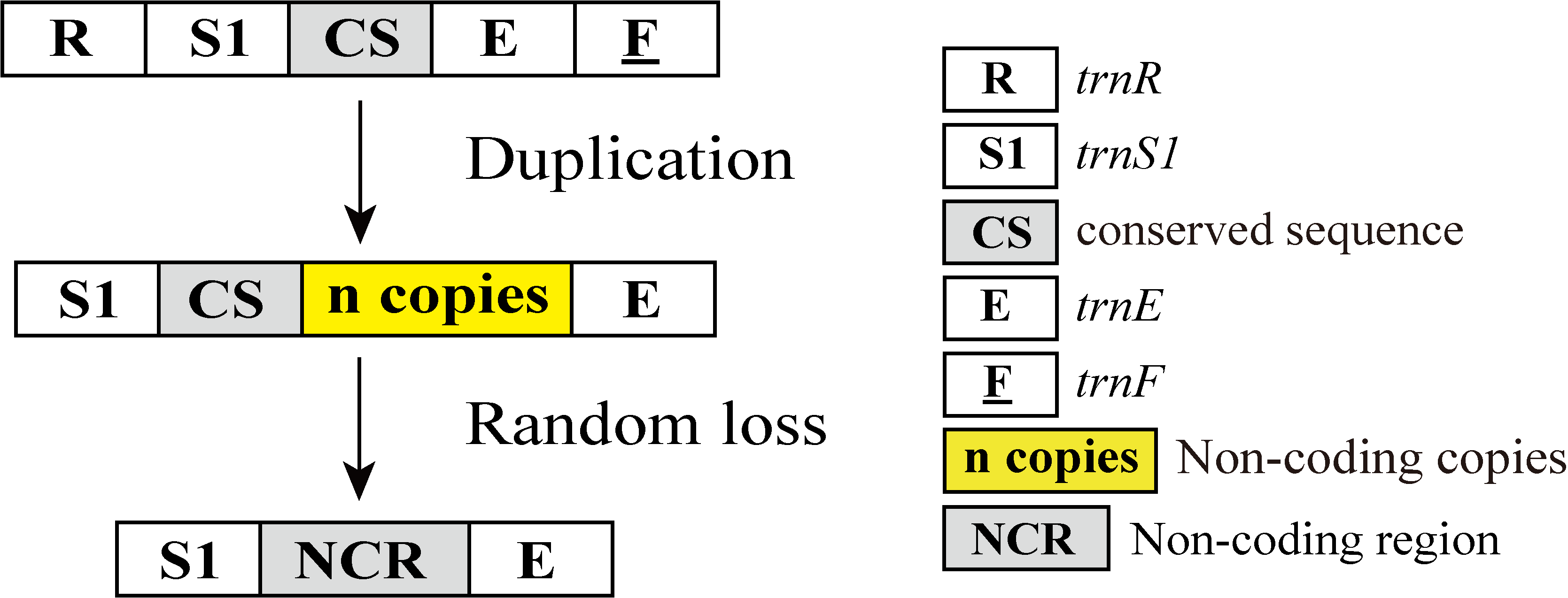

Supplement: Supplemental Information 17 — The duplication/random loss model can explain the NCR between trnS1 (AGN) and trnE. The CS indicates the 19 bp conserved sequence TTTTTAGCGAACTTAGGGG. Gene sizes are not drawn to scale. Genes located on the majority strand are shown along the top of the boxes whereas genes located on the minority strand are shown on the bottom. White boxes represent genes with the same relative position as in the ancestral insect arrangement pattern. Grey boxes represent non-coding regions. The remaining genes and gene orders that were identical to the ancestral insect are left out. [file peerj-08-9740-s017.png]
