# Supplementary material for: Six complete mitochondrial genomes of mayflies from three genera of Ephemerellidae (Insecta: Ephemeroptera) with inversion and translocation of trnI rearrangement and their phylogenetic relationships
Source: PeerJ. 2020 Aug 19;8:e9740. doi: 10.7717/peerj.9740 (PMC7443110; doi:10.7717/peerj.9740)
Supplement: Supplemental Information 18 [file peerj-08-9740-s018.pdf]

Table1 S1 Universal primers and specific primers used to amplify the mitogenomes of six mayfly mitochondrial genomes

Primers	Number	Primer name	Sequence (5'-3')	
Universal primers	1	FY-J-214	AAGCTHDTRGGTTCATAYCCY	Simon, 2006
		FY-N-1873	AANGGDGGRTAVACNGTYCA	Simon, 2006
	2	FY-J-1423	ACDAAYCAYAARGAYATYGG	Simon, 1994
		FY-N-2329	ACDGTRAAYATRTGRTGNGCYCA	Simon;1994
	3	FY-J-2198	TATTHTGATTYTTYGGNCAYCCHGAAGT	Simon, 2006
		FY-N-3705	GCYCCRCARATTTCNGAACATTG	Simon, 2006
	4	FY-J-4463	TTYGCHCAYYTDGTNCCNCARGG	Simon, 2006
		FY-N-5748	GGRTCRAANCCRCAYTCRAANGG	Simon, 2006
	5	FY-J-5747	CCATTYGAATGTGGATTTGAYCC	Simon, 2006
		FY-N-6160	YCAATTMTATCATTAACAGTGA	Simon, 2006
	6	FY-J-7077	AARTCCTTWGARTAAAAKCC	Simon, 2006
		FY-N-7793	TTRGGTWGRGATGGDTTRGG	Simon, 2006
	7	FY-J-7572	AAANGGRATYTGNGCDCTYTTHGT	Simon, 2006
		FY-N-8741	AYTTCRATNGYTTGHCCHT	Simon, 2006
	8	FY-J-8641	CNGAHGAACAHARNCCRTG	Simon, 2006
		FY-N-9629	GTHTGYGARGGAGCWYTKGG	Simon, 2006
	9	FY-J-10885	AYGTYCTRCCYTGRGGWCARATRTC	Simon, 1994
		FY-N-12964	TTACCTTARGGATAACAGCRTAW	Zhang et al., 2018
	10	FY-J-11335	CAYATYCARCCHGARTGRTA	Zhang et al. 2008
		FY-N-12965	TTACCTTAGGGATAACAGCRTWA	Zhang et al. 2018
	11	FY-J-12831	CGGTYTGAACTCAGATCATGTA	Simon, 1994
		FY-N-13889	KTACCTTKTGTATCAGGGTT	Simon, 2006
	12	FY-J-13286	CTTTGCACRGTCAVWATACYGC	Zhang et al., 2018
		FY-14722	GTGCCAGCVDCCGCGGTTANA	Simon, 2006
Specific primer	13	XIAOF89-J-14194	GTAGAATCCTCGGGGGTTAT	
		XIAOF89-N-571	TGGGAGCCAAAAGTGAAATG	
	14	XIAOF89-J-7429	CACTTTTTGTCATCGCAGC	
		XIAOF89-N-8659	AGTATTAGACTTGTCGGGGGAG	
	15	XIAOF89-J-8786	CTCTTACTGGGGCTTCTACA	
		XIAOF89-N-11083	GAGTGAGGATGGTCAGGATT	
	16	XIAOF89-J-3370	AGTTACGAGTATTCCGATTT	
		XIAOF89-N-4614	AAGGAGTAGGGCTATTTGTC	
	17	XIAOF89-J-5387	GAACACTTTTCACCTAACCAC	

	XIAOF89-N-7351	GCTTGTCCACTCTTCAACTCTA
18	XIAOF89-J-1133	CCGCCCTCGTTACTACCA
	XIAOF89-N-1813	AGACAGTCCACCCAGTTCCT
19	XIAOF89-J-14099	CCTTGACCTGACATACTCTC
	XIAOF89-N-367	TTTATCTGTTATTAGGGGGA
20	XIAOF94-J-3495	GTAGACAATCGTGTGGTGCT
	XIAOF94-N-4738	GGTGGTTATTATGTGCGGA
21	XIAOF94-J-8994	TGAGTAAGTGAAAGGGAACC
	XIAOF94-N-11218	AAGGATTAGCAGGGATGAAG
22	XIAOF94-J-5369	ACACTTCTCTCCCAATCATC
	XIAOF94-N-7062	AGGCTCTGCTTTTTATGTG
23	XIAOF94-J-7438	CCAATCAACCAATAATCTCC
	XIAOF94-N-8900	TTCCCTTTCACTTACTCACC
24	XIAOF94-J-14575	CATCCAGTTTAGTAGGGTCTC
	XIAOF94-N-751	GTGAAGAGCCTAAGCCTATC
25	XIAOF94-J-3495	GTAGACAATCGTGTGGTGCT
	XIAOF94-N-4738	GGTGGTTATTATGTGCGGA
26	XIAOF120-J-3487	ATAAAGACACAAATCCGAAT
	XIAOF120-N-4592	ACGAGTAAGAAAGAAACCAA
27	XIAOF120-J-5424	GCCGCCTGATACTGACATT
	XIAOF120-N-7282	GAAGGGACCATACCTGCTAAG
28	XIAOF120-J-7582	TACTCAAAGCCGTTAGCAT
	XIAOF120-N-8786	TGTGGAGGCACCAGTAAGA
29	XIAOF120-J-8782	AGAACCTCTTACTGGTGCCTC
	XIAOF120-N-10864	CAACAGCGAATCCTCCTCAT
30	XIAOF120-J-11159	TTACTCCTGTTCACATTCA
	XIAOF120-N-12808	CAGCGTAATCTTCTTTGAG
31	XIAOF120-J-14007	ACCCAAATCCACCTTCTCTC
	XIAOF120-N-371	TATTCTTACCCGAAAGCAGG
32	XIAOF137-J-1722	AATCTCAGCCATTCTATCG
	XIAOF137-N-2605	CTACATCCATCCCTACGG
33	XIAOF137-J-3523	GAGTTGTGTTGCCCATAAA
	XIAOF137-N-4804	TCAAAGTTGTTATCGCACC
34	XIAOF137-J-5621	TTACCATCACCGTTATCGTC
	XIAOF137-N-7153	GCAGGAGCAGTTATTCACAA

35	XIAOF137-J-7632	AGAATGAACGAGAGCAGATA
	XIAOF137-N-8923	GGTAGGAGGAGTTCTTGTTAG
36	XIAOF137-J-8965	TCACTCTTAGTTGTCGCCAG
	XIAOF137-N-11065	CCCAGTTTGGTGTAAGAATAG
37	XIAOF137-J-14442	TCTTCTACTACTCGGGACTCTT
	XIAOF137-N-787	ACGAAGAGAAGTTTGGTTGA

Note: The primers starting with XIAOF89, XIAOF94 and XIAOF137 are specific primers of *Ephemerella* sp. Yunnan-2018, *Serratella* sp. Yunnan-2018, *Serratella zapekinae* and *Serratella* sp. Liaoning-2019, respectively.
