# Supplementary material for: Six complete mitochondrial genomes of mayflies from three genera of Ephemerellidae (Insecta: Ephemeroptera) with inversion and translocation of trnI rearrangement and their phylogenetic relationships
Source: PeerJ. 2020 Aug 19;8:e9740. doi: 10.7717/peerj.9740 (PMC7443110; doi:10.7717/peerj.9740)
Supplement: Supplemental Information 19 [file peerj-08-9740-s019.pdf]

Table S2. Location of features in the mtDNA of *Ephemerella* sp. Yunnan-2018

Gene	Strand	Position	Length (nuc.)	Anti Codon	Start Codon	Stop Codon	Intergenic nucleotides
$tRNA^{Ile}$	-	1-66	66	ATC			0
CR	+	67-596	530				0
$tRNA^{Gln}$	-	597-665	69	CAA			0
$tRNA^{Met}$	+	665-728	64	ATG			-1
nad2	+	729-1751	1023		ATT	TAA	0
$tRNA^{Trp}$	+	1750-1817	68	TGA			-2
$tRNA^{Cys}$	-	1810-1870	61	TGC			-8
$tRNA^{Tyr}$	-	1871-1934	64	TAC			0
cox1	+	1906-3471	1566		ATC	TAA	-29
$tRNA^{Leu2}$	+	3467-3531	65	TTA			-5
cox2	+	3533-4240	708		ATG	TAA	+1
$tRNA^{Lys}$	+	4221-4289	69	AAG			-20
$tRNA^{Asp}$	+	4290-4355	66	GAC			0
atp8	+	4356-4520	165		ATA	TAA	0
atp6	+	4517-5191	675		ATA	TAA	-4
cox3	+	5191-5979	789		ATG	TAA	-1
$tRNA^{Gly}$	+	5982-6043	62	GGA			+2
nad3	+	6044-6397	354		TTG	TAG	0
$tRNA^{Ala}$	+	6396-6458	63	GCA			-2
$tRNA^{Arg}$	+	6458-6519	62	CGA			-1
$tRNA^{Asn}$	+	6517-6580	64	AAC			-3
$tRNA^{Ser1}$	+	6578-6644	67	AGC			-3
$tRNA^{Glu}$	+	6645-6708	64	GAA			0
$tRNA^{Phe}$	-	6707-6769	63	TTC			-2
nad5	-	6770-8506	1737		ATA	TAA	0
$tRNA^{His}$	-	8507-8570	64	CAC			0
nad4	-	8570-9916	1347		ATG	TAG	-1
nad4l	-	9910-10206	297		ATG	TAA	-7
$tRNA^{Thr}$	+	10275-10335	61	ACA			+68
$tRNA^{Pro}$	-	10336-10400	65	CCA			0
nad6	+	10403-10927	525		TTG	TAA	+2
cytb	+	10927-12105	1179		ATG	TAA	-1
$tRNA^{Ser2}$	+	12062-12130	69	TCA			-44
nad1	-	12148-13086	939		ATG	TAA	+17
$tRNA^{Leu1}$	-	13088-13152	65	CTA			+1
16S rRNA	-	13153-14375	1223				0
$tRNA^{Val}$	-	14376-14445	70	GTA			0
12S rRNA	-	14447-15222	776				+1
