# Supplementary material for: Six complete mitochondrial genomes of mayflies from three genera of Ephemerellidae (Insecta: Ephemeroptera) with inversion and translocation of trnI rearrangement and their phylogenetic relationships
Source: PeerJ. 2020 Aug 19;8:e9740. doi: 10.7717/peerj.9740 (PMC7443110; doi:10.7717/peerj.9740)
Supplement: Supplemental Information 20 — Orange shading represents Ephemerella sp. Yunnan-2018. Blue shading represents Serratella zapekinae. Yellow shading represents Serratella sp. Yunnan-2018. Green shading represents Serratella sp. Liaoning-2019. [file peerj-08-9740-s020.pdf]

Table S3. Location of features in the mtDNA of *Serratella zapekinae*

Gene	Strand	Position	Length (nuc.)	Anti Codon	Start Codon	Stop Codon	Intergenic nucleotides
$tRNA^{Ile}$	-	1-66	66	ATC			0
CR	+	67-1102	1039				0
$tRNA^{Gln}$	-	1103-1171	69	CAA			0
$tRNA^{Met}$	+	1172-1237	66	ATG			0
nad2	+	1238-2260	1023		ATT	TAA	0
$tRNA^{Trp}$	+	2259-2326	68	TGA			-2
$tRNA^{Cys}$	-	2319-2379	61	TGC			-8
$tRNA^{Tyr}$	-	2380-2446	67	TAC			0
cox1	+	2447-3983	1554		ATC	TAA	0
$tRNA^{Leu2}$	+	3979-4043	65	TTA			-5
cox2	+	4045-4732	688		ATG	T	+1
$tRNA^{Lys}$	+	4733-4801	69	AAG			0
$tRNA^{Asp}$	+	4802-4867	66	GAC			0
atp8	+	4868-5029	162		ATC	TAA	0
atp6	+	5026-5700	675		ATA	TAA	-4
cox3	+	5700-6488	789		ATG	TAA	-1
$tRNA^{Gly}$	+	6488-6550	63	GGA			-1
nad3	+	6551-6904	354		ATG	TAG	0
$tRNA^{Ala}$	+	6903-6967	65	GCA			-2
$tRNA^{Arg}$	+	6968-7029	62	CGA			0
$tRNA^{Asn}$	+	7027-7092	66	AAC			-3
$tRNA^{Ser1}$	+	7090-7156	67	AGC			-3
$tRNA^{Glu}$	+	7157-7222	66	GAA			0
$tRNA^{Phe}$	-	7221-7284	64	TTC			-2
nad5	-	7285-9025	1741		GTG	T	0
$tRNA^{His}$	-	9026-9089	64	CAC			0
nad4	-	9089-10435	1347		ATG	TAA	-1
nad4l	-	10429-10725	297		ATG	TAA	-7
$tRNA^{Thr}$	+	10728-10790	63	ACA			+2
$tRNA^{Pro}$	-	10791-10855	65	CCA			0
nad6	+	10858-11373	516		TTG	TAA	+2
cytb	+	11373-12507	1135		ATG	T	-1
$tRNA^{Ser2}$	+	12508-12576	69	TCA			0
nad1	-	12594-13532	939		ATT	TAA	+17
$tRNA^{Leu1}$	-	13533-13597	65	CTA			0
16S rRNA	-	13598-14820	1223				0
$tRNA^{Val}$	-	14821-14889	69	GTA			0
12S rRNA	-	14890-15660	771				0
