# Supplementary material for: Six complete mitochondrial genomes of mayflies from three genera of Ephemerellidae (Insecta: Ephemeroptera) with inversion and translocation of trnI rearrangement and their phylogenetic relationships
Source: PeerJ. 2020 Aug 19;8:e9740. doi: 10.7717/peerj.9740 (PMC7443110; doi:10.7717/peerj.9740)
Supplement: Supplemental Information 21 [file peerj-08-9740-s021.pdf]

Table S4. Location of features in the mtDNA of *Serratella* sp. Yunnan-2018

Gene	Strand	Position	Length (nuc.)	Anti Codon	Start Codon	Stop Codon	Intergenic nucleotides
tRNA^{Ile}	-	1-67	67	ATC			0
CR	+	68-526	459				0
tRNA^{Gln}	-	527-595	69	CAA			0
tRNA^{Met}	+	596-662	67	ATG			0
nad2	+	663-1685	1023		ATT	TAA	0
tRNA^{Trp}	+	1684-1751	68	TGA			-2
tRNA^{Cys}	-	1744-1805	62	TGC			-8
tRNA^{Tyr}	-	1806-1872	67	TAC			0
cox1	+	1835-3409	1575		ATT	TAA	-38
tRNA^{Leu2}	+	3405-3469	65	TTA			-5
cox2	+	3471-4158	688		ATG	T	+1
tRNA^{Lys}	+	4159-4227	69	AAG			0
tRNA^{Asp}	+	4228-4293	66	GAC			0
atp8	+	4294-4455	162		ATT	TAA	0
atp6	+	4452-5126	675		ATA	TAA	-4
cox3	+	5126-5914	789		ATG	TAA	-1
tRNA^{Gly}	+	5914-5975	62	GGA			-1
nad3	+	5976-6329	354		GTG	TAG	0
tRNA^{Ala}	+	6328-6391	64	GCA			-2
tRNA^{Arg}	+	6392-6455	64	CGA			0
tRNA^{Asn}	+	6453-6517	65	AAC			-3
tRNA^{Ser1}	+	6515-6581	67	AGC			-3
tRNA^{Glu}	+	6582-6645	64	GAA			0
tRNA^{Phe}	-	6644-6709	66	TTC			-2
nad5	-	6711-8453	1743		ATA	TAA	+1
tRNA^{His}	-	8451-8515	65	CAC			-3
nad4	-	8515-9861	1347		ATG	TAA	-1
nad4l	-	9855-10151	297		ATG	TAA	-7
tRNA^{Thr}	+	10157-10219	63	ACA			+5
tRNA^{Pro}	-	10220-10285	66	CCA			0
nad6	+	10291-10806	516		ATA	TAA	+5
cytb	+	10807-11943	1137		ATG	TAG	0
tRNA^{Ser2}	+	11943-12011	69	TCA			-1
nad1	-	12031-12969	939		GTG	TAA	+19
tRNA^{Leu1}	-	12970-13034	65	CTA			0
16S rRNA	-	13035-14264	1230				0
tRNA^{Val}	-	14265-14333	69	GTA			0
12S rRNA	-	14335-15112	778				+1
