# Supplementary material for: Six complete mitochondrial genomes of mayflies from three genera of Ephemerellidae (Insecta: Ephemeroptera) with inversion and translocation of trnI rearrangement and their phylogenetic relationships
Source: PeerJ. 2020 Aug 19;8:e9740. doi: 10.7717/peerj.9740 (PMC7443110; doi:10.7717/peerj.9740)
Supplement: Supplemental Information 22 [file peerj-08-9740-s022.pdf]

Table S5. Location of features in the mtDNA of *Serratella* sp. Liaoning-2019

Gene	Strand	Position	Length (nuc.)	Anti Codon	Start Codon	Stop Codon	Intergenic nucleotides
$tRNA^{Ile}$	-	1-67	67	ATC			0
CR	+	68-959	892				0
$tRNA^{Gln}$	-	960-1028	69	CAA			0
$tRNA^{Met}$	+	1029-1093	65	ATG			0
nad2	+	1094-2116	1023		ATT	TAA	0
$tRNA^{Trp}$	+	2115-2182	68	TGA			-2
$tRNA^{Cys}$	-	2175-2236	62	TGC			-8
$tRNA^{Tyr}$	-	2237-2302	66	TAC			0
cox1	+	2286-3839	1554		ATC	TAA	-17
$tRNA^{Leu2}$	+	3835-3899	65	TTA			-5
cox2	+	3901-4608	708		ATG	TAA	+1
$tRNA^{Lys}$	+	4589-4657	69	AAG			-20
$tRNA^{Asp}$	+	4658-4723	66	GAC			0
atp8	+	4724-4885	162		ATC	TAA	0
atp6	+	4882-5556	675		ATA	TAA	-4
cox3	+	5556-6344	789		ATG	TAA	-1
$tRNA^{Gly}$	+	6344-6405	62	GGA			-1
nad3	+	6412-6759	348		ATT	TAG	+6
$tRNA^{Ala}$	+	6758-6822	65	GCA			-2
$tRNA^{Arg}$	+	6824-6883	60	CGA			+1
$tRNA^{Asn}$	+	6882-6945	64	AAC			-2
$tRNA^{Ser1}$	+	6943-7009	67	AGC			-3
$tRNA^{Glu}$	+	7010-7073	64	GAA			0
$tRNA^{Phe}$	-	7072-7135	64	TTC			-2
nad5	-	7136-8876	1741		GTG	T	0
$tRNA^{His}$	-	8877-8940	64	CAC			0
nad4	-	8941-10285	1345		GTG	T	0
nad4l	-	10279-10575	297		ATG	TAA	-7
$tRNA^{Thr}$	+	10578-10642	65	ACA			+2
$tRNA^{Pro}$	-	10643-10707	65	CCA			0
nad6	+	10710-11226	517		TTG	T	+2
cytb	+	11227-12405	1179		ATG	TAA	0
$tRNA^{Ser2}$	+	12362-12430	69	TCA			-44
nad1	-	12448-13386	939		ATT	TAA	+17
$tRNA^{Leu1}$	-	13387-13451	65	CTA			0
16S rRNA	-	13452-14673	1222				0
$tRNA^{Val}$	-	14674-14743	70	GTA			0
12S rRNA	-	14744-15514	771				0
