# Supplementary material for: Six complete mitochondrial genomes of mayflies from three genera of Ephemerellidae (Insecta: Ephemeroptera) with inversion and translocation of trnI rearrangement and their phylogenetic relationships
Source: PeerJ. 2020 Aug 19;8:e9740. doi: 10.7717/peerj.9740 (PMC7443110; doi:10.7717/peerj.9740)
Supplement: Supplemental Information 23 [file peerj-08-9740-s023.pdf]

Table S6. Ephemeroptera species with the A+T content of the control region and whole mitogenome including six species involved in this study

Species	A+T content	
	Mitogenome	Control region
Paegniodes cupulatus	65.6	63.2
Parafronurus youi	66.4	57.0
Ephemerella sp. Yunnan-2018	61.1	54.5
Serratella zapekinae	65.2	61.9
Serratella sp. Yunnan-2018	66.1	62.1
Serratella sp. Liaoning-2019	65.2	68.5
Torleya grandipennis	61.2	61.1
Torleya tumiforceps	62.6	63.6
